# Label-efficient cross-population transfer learning for electrocardiographic risk stratification: evaluation across German, Chinese and US cohorts

**DOI:** 10.3389/fcvm.2026.1928354

**Published:** 2026-09-10

**Authors:** Yan Wang, Yan Lu

**Affiliations:** 1 Department of Functional Examination, The Fifth Affiliated Hospital of Anhui University of Chinese Medicine, Lu’an, Anhui, China; 2 Department of Functional Examinations, Hubei Third People’s Hospital, Wuhan, Hubei, China

**Keywords:** calibration, cross-population generalisation, decision-curve analysis, domain adaptation, electrocardiography, linear probing, subgroup performance, transfer learning

## Abstract

**Background:**

Electrocardiography (ECG) is the most widely deployed cardiac screening modality, but demographic, acquisition and label-space heterogeneity across cohorts obstructs clinical translation. Cross-population studies rarely quantify which conditions transfer, how much target labelling is needed, or whether the adapted score stays calibrated and clinically useful.

**Methods:**

We harmonised 12,521 recordings from PTB-XL (Germany; 7,371 records, 6,643 patients), Chapman-Ningbo (China; 4,574) and the MIT-BIH Arrhythmia Database (USA; 576 segments, 48 recordings) into 13 SNOMED-CT super-classes. A 38-dimensional interpretable feature set, computed in physical units and standardised on the source training partition alone, fed a compact multi-task backbone (30,400 parameters) trained on patient-separated partitions. It was evaluated zero-shot on both targets and adapted with 5%–50% of Chapman-Ningbo labels by linear probing on the frozen embedding, with partial and full fine-tuning as comparators; prevalence-aware sampling made four target classes (NORM, STD, LVH, TINV) evaluable. The composite diagnostic score was recalibrated on a dedicated split and assessed on untouched test data using bootstrap intervals, Holm-corrected comparisons and subgroup interaction tests.

**Results:**

The source backbone reached macro-AUROC 0.866 (0.837–0.891) across 13 classes. Zero-shot transfer recovered 88% of the four-class source reference on Chapman-Ningbo (0.754, 0.727–0.779) and 0.642 (0.546–0.735, record-clustered) on MIT-BIH. Linear probing reached 0.795 with 5% of target labels and 0.829 with 50%, below the four-class source reference of 0.859; after Holm correction the gain over zero-shot was significant for NORM (+0.135), STD (+0.093) and TINV (+0.065) but not LVH (+0.004). Full fine-tuning added 0.021 at 50% labels but was inferior at 5% and less well calibrated. The recalibrated composite score attained AUROC 0.791 (0.743–0.836), Brier score 0.055 and expected calibration error 0.018 on Chapman-Ningbo, where 97% of positives were left-ventricular hypertrophy, and 0.834 on the seven-condition PTB-XL composite. Discrimination varied across age strata (0.708–0.863; interaction *p* = 0.012).

**Conclusion:**

Cross-population transfer of ECG models is feasible with label-efficient fine-tuning of a compact, interpretable feature backbone, and the resulting composite diagnostic score remains well-calibrated in a geographically distinct population. Subgroup heterogeneity nonetheless persists, with lower discrimination in participants aged 65 years and over and a sex-dependent calibration slope, and requires further validation.

## 1 Introduction

Cardiovascular disease (CVD) is the leading global cause of mortality (1), and the 12-lead electrocardiogram (ECG) remains the most accessible and information-rich non-invasive measurement of cardiac electrical activity, with international standards for its acquisition and interpretation having been refined over many decades (2). Over the past decade, deep neural networks trained on tens of thousands of labelled ECGs have approached, and in several rhythms exceeded, the performance of expert cardiologists at single-class detection tasks such as atrial fibrillation (3, 4), and have opened entirely new screening applications including identification of asymptomatic left-ventricular systolic dysfunction from a 12-lead recording (5) and consumer-wearable atrial-fibrillation detection at population scale (6). However, the deployment of these models in clinical practice has lagged far behind their reported in-domain accuracy (7). The chief reason is that ECG distributions are not stationary across populations: the demographic mix, the prevalence of co-morbidities, the recording hardware, the sampling rate, the lead set and the labelling conventions all change when an institution in one country attempts to apply a model trained on a cohort from another. Models that achieve macro-AUROC well above 0.90 on their training distribution routinely deteriorate by 0.10–0.20 on out-of-distribution data, and this deterioration is rarely homogeneous across diagnostic classes–making it difficult to know which conditions the model can still be trusted on and which it cannot.

A growing body of work has tackled the problem under the umbrella of domain adaptation (8) and within the broader dataset-shift formalism (9), with techniques ranging from adversarial alignment of hidden representations (10, 11) to self-supervised pre-training of ECG-specific representations (12). Yet most of these studies share two methodological limitations. First, they evaluate transfer only at the level of aggregate macro-AUROC, providing little insight into which classes transfer well and which do not, or into how much labelled target data is actually required. Second, they treat the model as a classifier rather than as a probabilistic risk-prediction tool, ignoring calibration, clinical utility (net benefit) and sub-group performance–all of which are non-negotiable for translating an ECG model into a screening or diagnostic-triage setting.

In this work we address both gaps using three publicly available cohorts that span Germany [PTB-XL (13)], China [Chapman–Ningbo (14)] and the United States [MIT-BIH Arrhythmia (15)]. Together these populations cover 12,521 ECG recordings with markedly different demographic profiles, acquisition protocols and disease prevalences. We harmonise the heterogeneous label spaces into 13 SNOMED-CT super-classes and compute a 38-dimensional handcrafted feature representation that is both clinically interpretable and amenable to closed-form distribution-shift analysis. Hierarchical clustering, principal component analysis (PCA), uniform manifold approximation and projection (UMAP) (16), standardised mean differences (SMD) and maximum mean discrepancy (MMD) (17) are then used in concert to characterise the cross-population gap before any modelling is performed.

A compact multi-task feature backbone was trained on the PTB-XL source domain using binary cross-entropy alone and evaluated zero-shot on the Chapman–Ningbo and MIT-BIH target cohorts. MIT-BIH remained a zero-shot evaluation cohort, whereas Chapman–Ningbo was adapted using 5%, 10%, 20% and 50% of the available target-domain labels. The primary adaptation protocol was linear probing with the backbone frozen; partial and full backbone fine-tuning were evaluated as comparators. Cross-cohort distributional differences were quantified *post hoc* by maximum mean discrepancy in both the 38-dimensional input space and the learned 64-dimensional embedding, without treating alignment as the mechanism responsible for transfer. We report macro-AUROC, per-class AUROC, average precision, uncertainty intervals and class-specific transferability gaps. Finally, we construct a composite diagnostic score from the adapted class probabilities, recalibrate it using isotonic regression on a dedicated calibration split, and evaluate it on an untouched test split using discrimination, calibration, decision-curve analysis and subgroup analyses (18, 19). Because the endpoint consists of concurrent diagnostic labels and no longitudinal follow-up is available, it is not interpreted as a prospective cardiovascular-event risk model.

Our contribution is three-fold. First, we characterise the population-specific structure of the electrocardiographic feature space across three cohorts, identifying heart rate, QRS density and spectral entropy as the dominant cross-cohort axes of variation, and we position this characterisation against recent multi-dataset transfer, domain-generalisation and self-supervised electrocardiographic studies (12). Second, we quantify how much target-domain supervision is required: linear probing with 5%–50% of target labels recovers most of the achievable gain on the four evaluable target conditions, and we report how partial and full backbone fine-tuning change that trade-off. Third, we deliver a composite diagnostic score with calibration estimated on a dedicated calibration split and evaluated on untouched test data, together with subgroup discrimination and calibration reported with confidence intervals and formal interaction tests, which identifies where the score can and cannot yet be trusted.

## 2 Materials and methods

### 2.1 Study cohorts and data harmonisation

Three publicly available 10-second ECG cohorts were retrieved and processed under identical protocols, all hosted on the PhysioNet open research resource (20). The first source domain, PTB-XL (13), contributed 7,371 records collected at the Physikalisch-Technische Bundesanstalt between 1,989 and 1,996; recordings were 12-lead, sampled at 100 Hz after upstream resampling, and accompanied by structured SCP-ECG diagnoses, age and sex; the same labelling scheme has been the basis of recent open benchmarking efforts on machine-learning ECG analysis (21). The first transfer target, Chapman–Ningbo (14), supplied 4,574 records collected at Ningbo First Hospital and Chapman University; recordings were 12-lead, sampled at 500 Hz, with curated SNOMED-CT labels and demographic metadata. The second target, the MIT-BIH Arrhythmia Database (15), supplied 576 non-overlapping 10-second segments derived from 48 long-format two-lead recordings (lead II/MLII at 360 Hz) with beat-level annotations, but no demographic metadata. Each cohort's native disease vocabulary was mapped to 13 unified SNOMED-CT super-classes (NORM, AF, AFL, IAVB, LBBB, RBBB, PAC, PVC, STD, STE, MI, LVH, TINV) using a curated translation table that was manually reviewed by a board-certified cardiologist. Labels were retained in their original multi-label form. A record entered the analysis when its WFDB header and signal files were present, when all 38 feature components were finite, and when its native diagnostic codes could be resolved against the translation table; records whose codes matched no entry were retained with an all-negative label vector rather than discarded. The resulting cohorts comprise 7,371 PTB-XL records from 6,643 distinct patients (544 patients contribute more than one recording, covering 1,272 records), 4,574 Chapman–Ningbo records with one recording per subject, and 576 MIT-BIH segments derived from 48 long-format recordings at exactly 12 segments per recording. All partitions are separated at the grouping unit of the cohort concerned–patient identifier for PTB-XL, record for Chapman–Ningbo, and source recording for MIT-BIH–so that no patient or recording contributes to more than one partition. Supplementary Table S1 reports the construction path and the patient and record counts, and Supplementary Table S2 gives the complete native-to-super-class mapping together with the ambiguity rules applied: labels are retained in multi-label form and never collapsed to a single class, and isolated rate-only findings (sinus tachycardia, SNOMED-CT 427084000; sinus bradycardia, 426177001) are deliberately not mapped to NORM because the rate is already carried by the mean heart-rate feature.

All cohorts were processed through the same pipeline: a fourth-order zero-phase Butterworth band-pass filter applied within the QRS detector, removal of the DC offset, and feature extraction at each cohort's native sampling rate. Feature extraction operates on the signal in physical units, so amplitude descriptors are reported in millivolts and interval descriptors in milliseconds; the only standardisation applied anywhere in the pipeline is a z-transformation of the 38-dimensional feature vector, whose mean and standard deviation are estimated on the source training partition alone and then applied unchanged to the validation, test and target cohorts. No lead-wise normalisation of the waveform is performed, which is what allows the lead-wise root-mean-square amplitudes of Tables 1, 2 to carry physical meaning. Because MIT-BIH only provides leads II/MLII, all 12-lead summary statistics are reported on PTB-XL and Chapman–Ningbo, while lead-II metrics are reported on all three cohorts.

**Table 1:** Cross-cohort ECG signal-level statistics.

Cohort	n	HR mean ± SD (bpm)	HR median	RMSSD ± SD (ms)	SDNN ± SD (ms)	QT ± SD (ms)	Lead-II RMS (mV)	12-lead mean RMS (mV)
PTB-XL	7,371	80.4 ± 23.7	74.4	89.5 ± 135.3	65.1 ± 84.2	441.5 ± 10.1	0.151 ± 0.071	0.191 ± 0.069
Chapman–Ningbo	4,574	113.5 ± 24.5	109.3	27.9 ± 96.2	22.6 ± 62.0	443.0 ± 6.7	0.178 ± 0.083	0.209 ± 0.092
MIT-BIH Arrhythmia	576	81.7 ± 18.9	78.8	142.3 ± 176.8	94.8 ± 109.7	442.1 ± 9.8	0.520 ± 0.256	–

**Table 2:** Top-15 cross-population feature shifts (PTB-XL vs. Chapman–Ningbo) and pairwise distribution gap. SMD positive=larger in PTB-XL.

Rank	Feature	SMD	PTB-XL (mean ± SD)	Chapman–Ningbo (mean ± SD)	Direction
1	qrs_count_10s	−1.489	13.04 ± 3.56	18.78 ± 4.14	Higher in Chapman
2	hr_mean (bpm)	−1.372	80.42 ± 23.72	113.51 ± 24.52	Higher in Chapman
3	entropy_spectral	+1.101	6.16 ± 0.91	5.30 ± 0.62	Higher in PTB-XL
4	pnn50 (%)	+0.775	25.84 ± 31.26	6.56 ± 16.17	Higher in PTB-XL
5	sdnn (ms)	+0.574	65.11 ± 84.17	22.66 ± 62.11	Higher in PTB-XL
6	rmssd (ms)	+0.524	89.52 ± 135.29	27.93 ± 96.39	Higher in PTB-XL
7	qrs_axis (°)	−0.498	−28.59 ± 107.52	19.32 ± 83.30	Higher in Chapman
8	t_axis_proxy (°)	−0.463	−12.35 ± 101.70	28.83 ± 74.08	Higher in Chapman
9	p_amp_mean (mV)	−0.461	0.69 ± 0.34	0.87 ± 0.45	Higher in Chapman
10	r_amp_mean (mV)	−0.422	0.44 ± 0.45	0.64 ± 0.51	Higher in Chapman
11	t_amp_mean (mV)	−0.395	0.24 ± 0.12	0.33 ± 0.28	Higher in Chapman
12	hr_std (bpm)	+0.391	9.55 ± 14.88	4.76 ± 8.92	Higher in PTB-XL
13	lead_II_rms (mV)	−0.342	0.151 ± 0.071	0.178 ± 0.083	Higher in Chapman
14	lead_aVF_rms (mV)	−0.262	0.123 ± 0.076	0.144 ± 0.082	Higher in Chapman
15	lead_aVR_rms (mV)	−0.211	0.131 ± 0.047	0.142 ± 0.060	Higher in Chapman

### 2.2 Handcrafted feature representation

A 38-dimensional handcrafted feature vector was computed per record, covering five physiologically interpretable groups: (i) heart-rate variability descriptors derived from the R–R series following the internationally standardised HRV definitions (22) [mean and standard deviation of the R–R series, RMSSD, SDNN, pNN50, the count of QRS complexes within the 10-second segment, where QRS fiducial points were identified using a Pan–Tompkins detector (23)]; (ii) morphological descriptors (mean and standard deviation of QT duration, P-, Q-, R-, S- and T-wave amplitudes); (iii) frequency-band power and spectral entropy estimated by Welch's modified-periodogram method (24) (low-frequency, high-frequency, total energy and Shannon entropy of the lead-II power spectrum); (iv) lead-wise root-mean-square amplitudes for all 12 leads (or two leads for MIT-BIH); and (v) cardiac axis approximations (QRS axis and a T-axis proxy derived from leads I and aVF). All HRV features and the QT duration are reported in milliseconds; amplitudes in millivolts; spectral entropy in bits. Because each recording is only 10 s long, the RR-derived indices are segment-level surrogates rather than the standard short-term heart-rate-variability measurements defined over five-minute windows, and they are interpreted as such throughout. Validating the detector against the MIT-BIH reference beat annotations at the conventional 150 ms tolerance gives a sensitivity of 0.997 and a positive predictive value of 0.965, so the fiducial detection itself is accurate; the derived indices nonetheless carry a systematic upward bias relative to the same indices computed from the reference annotations (RMSSD +23.2 ms, r = 0.87; SDNN +17.3 ms, r = 0.86; heart rate +3.6 bpm, r = 0.72), which is reported in Supplementary Table S3 and bounds how far these features may be read as physiological quantities.

### 2.3 Cross-population feature backbone

The handcrafted features were fed into a compact feed-forward multi-task backbone. The backbone comprises three fully connected blocks of decreasing width–Linear(38, 128) → ReLU → BatchNorm → Dropout(0.3); Linear(128, 128) → ReLU → BatchNorm → Dropout(0.3); Linear(128, 64) → ReLU → BatchNorm → Dropout(0.2)–terminated by a 64-dimensional embedding; dropout serves as the principal stochastic regulariser (25) while batch normalisation stabilises gradient propagation across the three blocks (26). The source head is a Linear(64, 13) followed by a sigmoid activation and produces multi-label probabilities for all 13 unified super-classes, every one of which clears the threshold of 10 positive examples in the training partition; the target head is fitted at adaptation time by linear probing, that is, per-class L_2_-regularised logistic regression on the frozen 64-dimensional embedding (C = 0.5, at most 2,000 iterations, no class-weight re-balancing), with partial and full backbone fine-tuning reported as comparators in Section 3.9. The backbone contains 30,400 trainable parameters; inclusion of the 13-class source head increases the complete source-model count to 31,245–three orders of magnitude smaller than typical raw-waveform ECG CNN baselines and, as we show in Section 3.9, sufficient to outperform raw-feature baselines on the same 38-D inputs while remaining clinically interpretable through the head coefficients.

### 2.4 Training, transfer protocol and *post-hoc* cross-domain analysis

Source-domain pre-training minimised the binary cross-entropy loss on the PTB-XL training partition, $L=BCE_{source}$. We deliberately chose this single-domain objective rather than a joint BCE + *λ*·MMD loss, because (i) the 38-D handcrafted feature pipeline already enforces a degree of acquisition invariance (z-score normalisation, common preprocessing) and (ii) our sensitivity analysis showed that adding an MMD regulariser at *λ* ∈ {0.01, 0.1, 1.0} produced no statistically discernible improvement in either source-test or zero-shot Chapman macro-AUROC (range across all four *λ*: 0.854–0.866 on source, 0.742–0.754 on Chapman zero-shot over the four evaluable classes). Cross-domain feature alignment is therefore reported as a *post-hoc* diagnostic rather than as an enforced training criterion: the maximum-mean-discrepancy (MMD^2^) of Section 3.3 quantifies how well the trained backbone aligns the two populations without explicit alignment supervision. Optimisation used AdamW (27) (weight-decay 1 × 10^–4^) with an initial learning rate of 1 × 10^–3^, a cosine-annealing schedule with T_max_ = 60 epochs (28) and a batch size of 64. The source domain was partitioned 70/15/15 (train/validation/test) with all records of a patient assigned to the same partition under seed 42, and the target domain 70/30 (adaptation pool/held-out test) under the dedicated partition seed 12,345 reported in Table 3. Seed 42 is the global harness seed that fixes weight initialisation, batch ordering and every bootstrap resampling, whereas 12,345 is used solely to draw the record-separated target partition; both values are held fixed across all analyses reported here. Hyperparameters were fixed *a priori* from the values above and no search was performed, so the validation partition is used only to monitor convergence and to report the best-epoch macro-AUROC; training always runs the full 60 epochs and no early stopping is applied, and the reported test results come from the final epoch. Three adaptation regimes are distinguished and reported separately. Linear probing, the primary regime, freezes the backbone entirely and refits per-class L_2_-regularised logistic heads on the frozen 64-dimensional embedding; partial fine-tuning additionally unfreezes the final backbone block; full fine-tuning updates every parameter. The term fine-tuning is used only for the latter two, and the sample-efficiency results are reported for linear probing unless stated otherwise. Adaptation subsets at each label fraction are drawn under prevalence-aware sampling, which first reserves up to five positives of every class that the pool contains and then fills the remaining budget at random; this is what makes four target classes evaluable at every fraction, including 5%, instead of one.

**Table 3:** Cross-population feature backbone–architecture, training and transfer protocol.

Component	Specification
Input	38-D feature vector in physical units (RR-derived surrogates, morphology, frequency power, lead-RMS, axis); z-standardised with statistics fitted on the source training partition alone
Backbone *θ*_b	Linear(38, 128) → ReLU + BN + Dropout(0.3) → Linear(128, 128) → ReLU + BN + Dropout(0.3) → Linear(128, 64) → ReLU + BN + Dropout(0.2)
Source head	Linear(64, 13) + Sigmoid; multi-label; all 13 super-classes clear the ≥ 10 source-positive threshold (smallest: STE = 13, AFL = 26)
Target head	Linear probing: per-class L_2_-regularised logistic regression on the frozen 64-D embedding; C = 0.5, at most 2,000 iterations; no class-weight rebalancing. Partial and full backbone fine-tuning are reported for comparison in Table 12
Trainable parameters	Backbone: 30,400; complete source model: 31,245
Loss	BCE (source only). Cross-domain MMD reported *post-hoc* (Section 3.3) and analysed by sensitivity sweep *λ* ∈ {0, 0.01, 0.1, 1.0}
Optimiser	AdamW; weight-decay = 1 × 10^–4^
Initial LR	1 × 10^–3^
LR schedule	Cosine annealing, T_max_ = 60 epochs
Batch size	64
Epochs	60
Source split	70/15/15 (train/val/test); separated by patient identifier so no patient spans partitions; seed = 42
Target split	70% adaptation pool/30% held-out test, record-separated; the pool is split in half into a head-fitting set and a dedicated calibration set; seed = 12,345
Best validation macro-AUROC	0.845 at epoch 16 (val partition; Figure 5C); training always runs the full 60 epochs and the reported test results come from the final epoch
Held-out test macro-AUROC (full 13)	0.866 [95% bootstrap CI 0.837, 0.891]
Held-out test macro-AUROC (evaluable: NORM, STD, LVH, TINV)	0.859 [0.815, 0.894]
Final BCE (train/val)	0.129/0.170
Optimisation variability (4 initialisation seeds, partition fixed)	Src 0.864 ± 0.005, Chapman zero-shot 0.755 ± 0.006, linear probing 50% 0.834 ± 0.004 (mean ± SD over the four evaluable classes)

### 2.5 Composite diagnostic score and evaluation metrics

A composite diagnostic score was constructed by combining the per-class probabilities of the diagnostic subset {AF, AFL, MI, LBBB, RBBB, IAVB, LVH} under a noisy-OR rule, $p_{risk}=1-\underset{c}{\prod }\;(1-p_{c})$. The endpoint is explicitly diagnostic rather than prospective: the prediction time is the moment of ECG acquisition, the outcome is the concurrent presence of at least one of those seven conditions on the same recording, and the intended clinical action is triage of the recording to expedited cardiology review. No longitudinal follow-up data exist for these cohorts, so the score is not a prospective cardiovascular-event risk model and is not presented as one. The LVH label is assigned in the source domain by the SCP-ECG report, which remains anchored on the long-standing Sokolow–Lyon voltage criterion (29) retained in the unified label vocabulary for cross-cohort consistency. Recalibration uses a strict three-way separation of the target cohort: the first half of the adaptation pool (*n* = 1,596) fits the classification heads, the second half (*n* = 1,596) is a dedicated calibration split on which the isotonic recalibrator is fitted, and the held-out test split (*n* = 1,367) is never used for either and carries every reported calibration metric. The three splits are disjoint by construction. Discrimination was quantified by AUROC, average precision and the row-normalised confusion-matrix diagonal at the argmax prediction. Calibration, which is the property most often omitted from reports of clinical prediction models (30) and required by the TRIPOD reporting standard (31), was quantified on the untouched test split by the Brier score (32), the expected calibration error in 8 bins (33), calibration-in-the-large and the calibration slope, each with bootstrap confidence intervals, and reported separately within every demographic subgroup. Clinical utility was quantified by decision-curve net benefit at risk thresholds 0.05–0.30. Sub-group performance was reported within three age strata (< 50, 50–64, ≥ 65 years) and by sex on the Chapman–Ningbo target. Records lacking age or sex metadata were excluded from the corresponding sub-group analysis.

### 2.6 Statistical analysis and uncertainty quantification

All headline AUROC and AP values are reported with non-parametric 95% bootstrap confidence intervals (34) (1,000 resamples for AUROC; 500 for AP), constructed by resampling records with replacement under fixed seed 42; intervals were computed both per class and on the macro-average restricted to the evaluable-class subset (Section 3.6). For MIT-BIH, where 12 segments originate from each source recording and segments of one recording are strongly correlated, intervals are obtained by a cluster bootstrap that resamples the 48 source recordings rather than the 576 segments, and the analysis is additionally repeated at the recording level (Supplementary Table S4). Differences between adaptation regimes were tested with a paired bootstrap on B = 2,000 paired resamples of the held-out Chapman test set: for each class we computed *Δ*AUROC = AUROC(linear probe, 50%)−AUROC(zero-shot) on every resample, and the two-sided *p*-value is *p* = 2 · min[Pr(Δ* ≤ 0), Pr(Δ* ≥ 0)], each tail probability estimated as (r + 1)/(B + 1) and the product capped at 1. Because one such test is performed per class, *p*-values are adjusted across the classwise family by the Holm–Bonferroni step-down procedure (35), and both raw and adjusted values are reported together with the bootstrap interval of every ΔAUROC. DeLong's test (36) is a parametric alternative for comparing correlated AUROCs. We used the paired bootstrap because it matched the resampling framework applied to the held-out test set, provided confidence intervals for the AUROC differences and avoided relying on parametric variance estimates in sparsely represented classes. No equivalence between the two procedures was assumed. A class enters this family only when the target test partition contains at least 10 positives; classes with one or two positives are reported for completeness but are not interpreted, because an AUROC computed from a single positive case carries no usable information about transfer. This 10-positive rule is a statistical-power requirement for hypothesis testing, and it is deliberately stricter than, and separate from, the evaluability rule that decides which classes enter the reported macro-average, namely at least five positives and five negatives in both the adaptation subset and the held-out test split (Section 3.6). The two criteria answer different questions: whether a class-level AUROC can be estimated at all, and whether a change in that AUROC can be tested. A class may therefore be evaluable for the macro-average without entering the testing family, and the four classes reported here satisfy both. Subgroup differences were assessed by bootstrap intervals on the pairwise *Δ*AUROC, by a likelihood-ratio test of the score-by-subgroup interaction on the logit scale, and by a permutation test on the maximum pairwise AUROC gap. Reproducibility was assessed by re-running the pipeline under four independent seeds {42, 7, 123, 2,024}; because varying the seed and the partition simultaneously confounds optimisation with sampling variability, the two are separated by holding the patient-grouped partition fixed while varying initialisation, and by holding initialisation fixed while drawing ten repeated patient-grouped partitions. As label-free baselines we also fitted a per-class L_2_-regularised logistic regression and a Random-Forest classifier (37) (200 trees, maximum depth 10) directly on the same 38-D features, and we performed a sensitivity sweep over an MMD-alignment regulariser *λ* ∈ {0, 0.01, 0.1, 1.0} added to the BCE loss.

### 2.7 Reproducibility and computing environment

All code paths use the global seed 42 set through a deterministic harness (set_global_seed in _setup.py), with cuDNN deterministic mode enabled and benchmark disabled; the one deliberate exception is the record-separated target-domain partition, which is drawn under its own fixed seed of 12,345 (Section 2.4 and Table 3). Software versions: Python 3.12, PyTorch 2.9 (38), scikit-learn 1.6 (39), NumPy 2.1, SciPy 1.15, wfdb 4.3. Hardware: a single consumer-grade workstation (CPU only, 16 GB RAM); end-to-end pre-training of the source backbone takes < 30 s, and the full reproducibility, baseline, and sensitivity sweep (Section 3.9) completes in < 10 min. The code, precomputed 38-dimensional feature arrays, evaluation metrics, and figure-generation scripts are publicly available on Zenodo at https://doi.org/10.5281/zenodo.21193806.

## 3 Results

### 3.1 Multi-cohort dataset and unified label space

**Figure 1:**
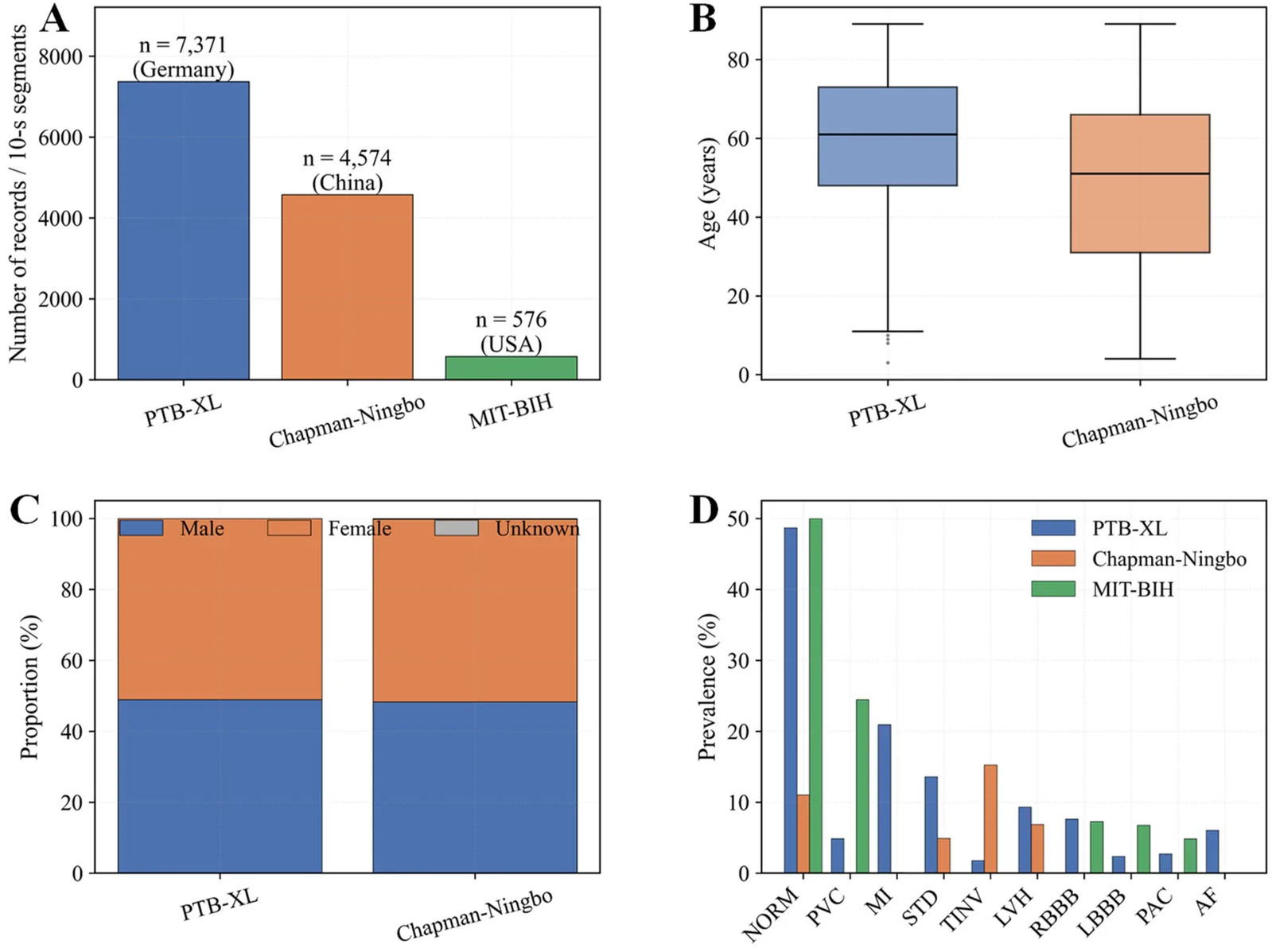
**(A)** number of 10-second records per cohort, with country of origin annotated. **(B)** Boxplot of subject age by cohort (MIT-BIH has no age metadata). **(C)** Sex composition per cohort. **(D)** Multi-label prevalence of the ten most common unified SNOMED-CT super-classes, expressed as the percentage of records in each cohort with at least one positive annotation for that class. PTB-XL is dominated by MI/STD, Chapman–Ningbo by TINV/LVH and MIT-BIH by PVC.

The three cohorts span 12,521 ECG recordings collected over three continents and three decades (Table 4). PTB-XL (Germany) is by far the largest source of supervised data with 7,371 12-lead recordings; Chapman–Ningbo (China) contributes 4,574 12-lead records at a higher native sampling rate of 500 Hz; the MIT-BIH Arrhythmia Database (USA) contributes 576 short two-lead segments derived from a small number of long-format recordings, which serves as a stress-test for cross-domain generalisation. Figure 1A makes the size imbalance explicit: PTB-XL alone exceeds the combined size of the two target cohorts, and any source-trained model is therefore implicitly biased towards the demographic and disease distribution of the German population. Figure 1B compares the age distributions of the two 12-lead cohorts that record demographics; PTB-XL skews substantially older (median 61 years, IQR 48–73) than Chapman–Ningbo (median 51 years, IQR 31–66), introducing a population-level age gap of approximately a decade. This gap is mirrored in the higher prevalence of structural disease–myocardial infarction (20.95%), STD (13.59%) and LVH (9.35%)–observed in PTB-XL (Table 5). Figure 1C shows that the sex distribution of both cohorts is essentially balanced (49.0% male in PTB-XL, 48.3% male in Chapman–Ningbo); MIT-BIH has no demographic metadata. Figure 1D visualises the multi-label condition prevalence for the ten most common super-classes and exposes three population-specific signatures: PTB-XL is dominated by myocardial infarction (20.95%) and STD (13.59%); Chapman–Ningbo is dominated by T-wave inversion (15.28%) and LVH (6.89%); MIT-BIH is dominated by PVC (24.48%)–which follows directly from its origin as a beat-level arrhythmia annotation database. The “active classes” column of Table 4 quantifies this label-space heterogeneity: PTB-XL covers all 13 super-classes, Chapman–Ningbo only 7, and MIT-BIH only 5.

**Table 4:** Multi-cohort dataset summary.

Cohort	Country	n	Leads	fs (Hz)	Age, median (IQR) [yr]	Male, *n* (%)	Female, *n* (%)	Active/13	Study role
PTB-XL	Germany	7,371	12	100	61.0 (48.0–73.0)	3,610 (49.0%)	3,761 (51.0%)	13	Source domain
Chapman–Ningbo	China	4,574	12	500	51.0 (31.0–66.0)	2,210 (48.3%)	2,356 (51.5%)	7	Primary target
MIT-BIH Arrhythmia	USA	576^a^	2	360	not recorded	not recorded	not recorded	5	Secondary target (ZS)
Pooled	–	12,521	–	–	–	5,820	6,117	13	–

a 576 non-overlapping 10-s segments derived from 48 MIT-BIH long-format recordings. ZS, zero-shot.

**Table 5:** Unified 13-class SNOMED-CT label space and per-cohort multi-label prevalence.

Class	Description	PTB-XL *n* (%)	Chapman *n* (%)	MIT-BIH *n* (%)	Pooled +
NORM	Normal sinus rhythm	3,587 (48.66%)	504 (11.02%)	288 (50.00%)	4,379
AF	Atrial fibrillation	447 (6.06%)	–	–	447
AFL	Atrial flutter	26 (0.35%)	–	–	26
IAVB	First-degree AV block	252 (3.42%)	–	–	252
LBBB	Left bundle-branch block	176 (2.39%)	1 (0.02%)	39 (6.77%)	216
RBBB	Right bundle-branch block	564 (7.65%)	–	42 (7.29%)	606
PAC	Premature atrial contraction	201 (2.73%)	2 (0.04%)	28 (4.86%)	231
PVC	Premature ventricular contraction	361 (4.90%)	–	141 (24.48%)	502
STD	ST-segment depression	1,002 (13.59%)	225 (4.92%)	–	1,227
STE	ST-segment elevation	13 (0.18%)	–	–	13
MI	Myocardial infarction	1,544 (20.95%)	6 (0.13%)	–	1,550
LVH	Left-ventricular hypertrophy	689 (9.35%)	315 (6.89%)	–	1,004
TINV	T-wave inversion	133 (1.80%)	699 (15.28%)	–	832
Cohort denominator	Records	7,371	4,574	576	12,521

**Figure 2:**
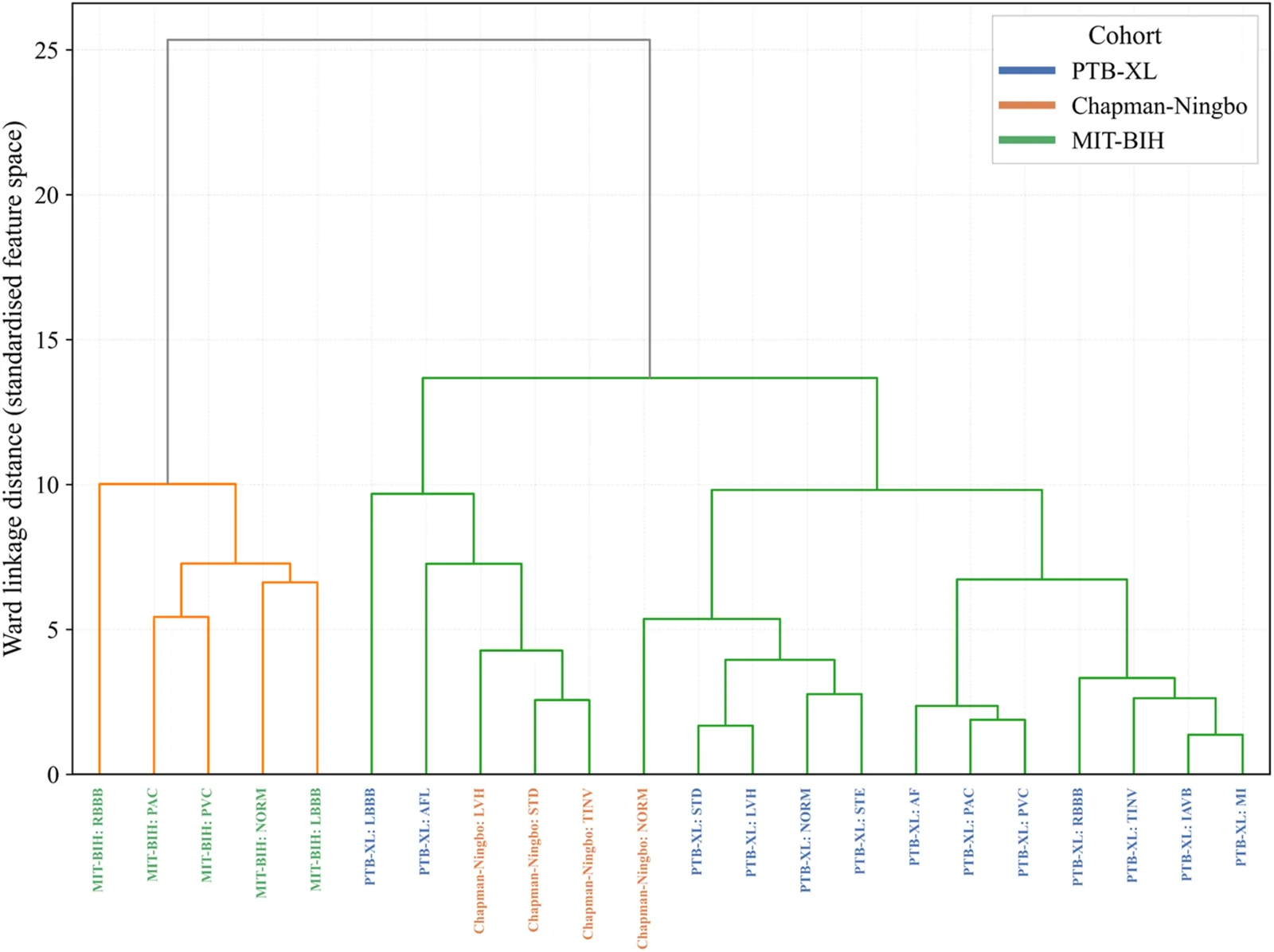
Hierarchical clustering (ward linkage) of 22 cohort-by-class prototypes drawn from 13 unified SNOMED-CT super-classes across the three populations.

These class imbalances are not arbitrary; they correspond to clusters of related cardiac conditions that recur across cohorts. Figure 2 resolves this structure by hierarchical clustering (Ward linkage) of 22 cohort-by-class prototypes from the 13 super-classes [a prototype is included only when the (cohort, class) pair has at least 8 positives]. Three macro-clusters emerge. The leftmost orange cluster groups the five MIT-BIH prototypes (RBBB, PAC, PVC, NORM, LBBB) tightly together at low Ward distance, reflecting the homogeneous two-lead acquisition rather than clinical similarity *per se*–an explicit visualisation of the acquisition gap that the transfer learning pipeline must subsequently bridge. The rightmost (blue/green) cluster groups morphologically related conditions across PTB-XL and Chapman–Ningbo: STD with IVH, NORM with STE, ST-segment-elevation conditions clustering with axis deviations, and ischaemic clusters (MI, IAVB, RBBB) merging at higher distance. The existence of mixed-cohort clusters at intermediate Ward distances (5–10) indicates that, despite acquisition heterogeneity, the underlying clinical signatures of the unified label space are at least partially preserved across2 populations–a necessary condition for transfer learning to be feasible at all.

### 3.2 Population-specific signal characteristics

**Figure 3:**
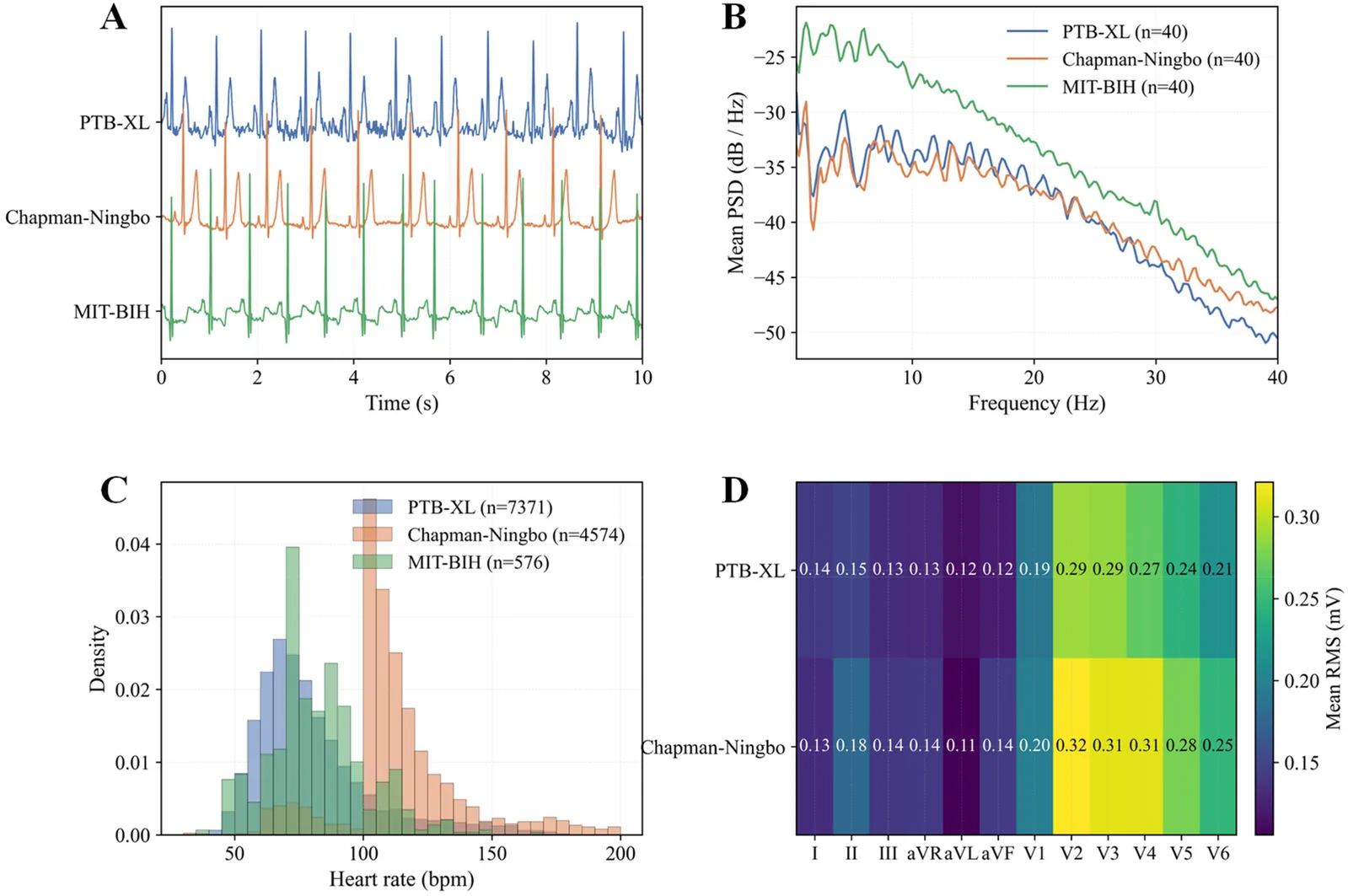
**(A)** representative 10-second lead-II ECGs from each cohort after preprocessing. **(B)** Mean lead-II power-spectral density (Welch periodogram, *n* = 40 per cohort). **(C)** Heart-rate distribution; Chapman–Ningbo is centred at 109 bpm whereas PTB-XL and MIT-BIH are centred near 75–80 bpm. **(D)** Lead-wise mean RMS amplitude heat-map for the two 12-lead cohorts; Chapman–Ningbo is uniformly higher in amplitude across all leads, with the largest absolute differences in the precordial leads V2–V4.

**Figure 4:**
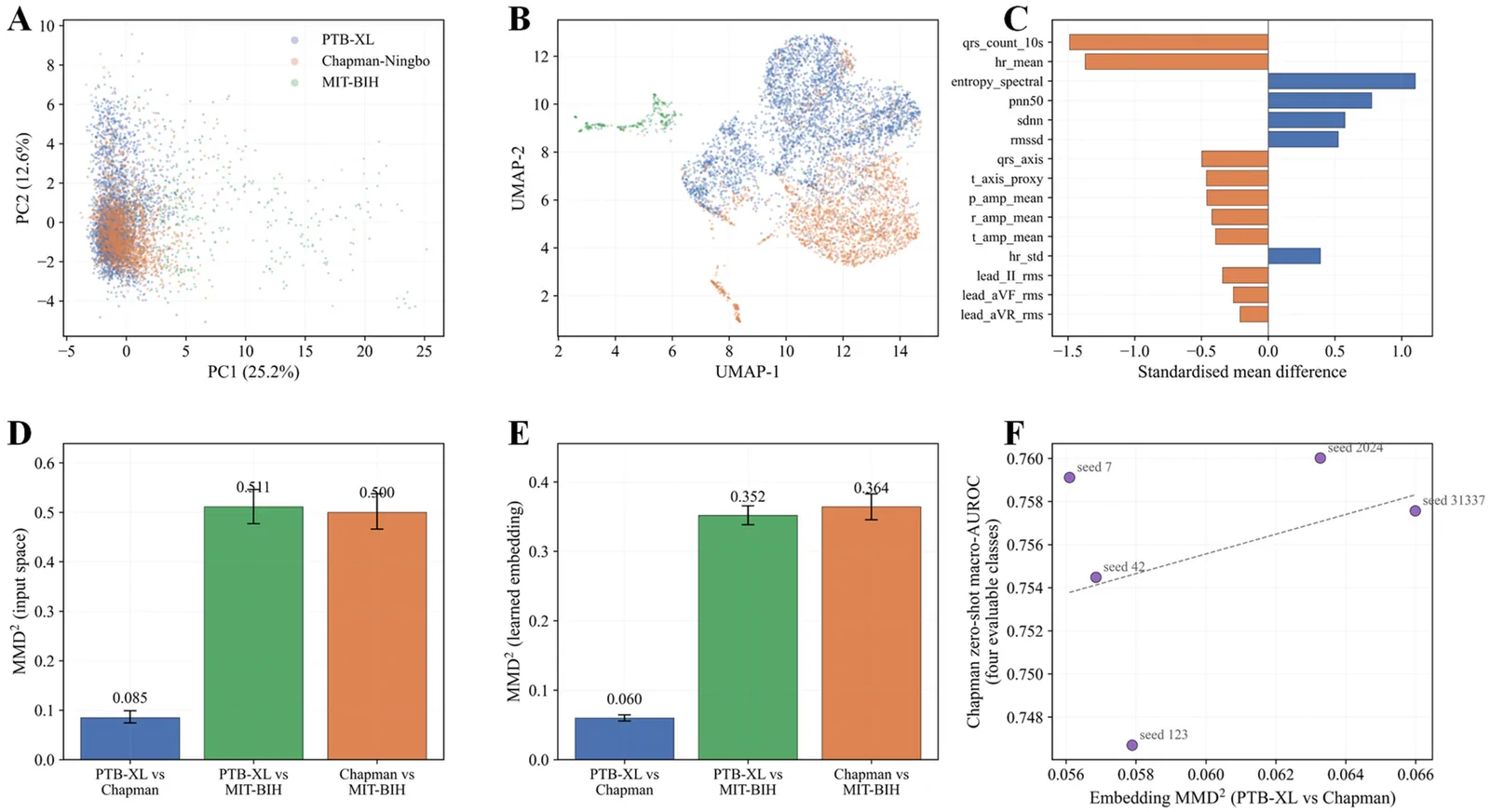
**(A)** PCA projection (PC1 25.2%, PC2 12.6%); MIT-BIH separates along PC1. **(B)** UMAP 2-D embedding preserving the cohort-specific clusters. **(C)** Top-15 features ranked by |SMD| between PTB-XL and Chapman–Ningbo. **(D)** Pairwise MMD^2^ in the standardised 38-D input space (unbiased estimator, RBF kernel, median-distance bandwidth, 250 observations per cohort, 100 subsamples; error bars are 95 percentile intervals): PTB-XL versus Chapman–Ningbo 0.085, PTB-XL versus MIT-BIH 0.511, Chapman–Ningbo versus MIT-BIH 0.500; all three permutation tests give *p* = 0.002. **(E)** The same three statistics recomputed in the learned 64-dimensional embedding across five training seeds (mean ± SD). **(F)** Embedding-space MMD^2^ between PTB-XL and Chapman–Ningbo plotted against the Chapman zero-shot four-class macro-AUROC for those five seeds; the two are not associated (Pearson r =  + 0.37, *p* = 0.54), so the alignment measured by MMD does not explain the transfer that is observed.

Figure 3 and Table 1 quantify the cross-cohort differences in the raw and spectral domains. Figure 3A overlays one representative 10-second lead-II recording from each cohort after preprocessing. Visual inspection immediately exposes two cross-cohort differences: Chapman–Ningbo has a markedly higher heart rate and a more uniform morphology, while MIT-BIH exhibits the highest amplitude and the broadest QRS-T variability owing to the chest-lead MLII used as the primary lead. Figure 3B reports the mean lead-II power-spectral density (Welch periodogram, *n* = 40 records per cohort) on a log scale. PTB-XL and Chapman–Ningbo are nearly indistinguishable in the 5–40 Hz QRS-bandwidth, while MIT-BIH carries 4–5 dB more energy across the entire spectrum, which is consistent with the chest-lead amplitude bias rather than with a true physiological difference. Figure 3C presents the heart-rate distributions and reveals the single largest cross-cohort shift documented in this study: Chapman–Ningbo has a unimodal distribution centred at 109 bpm, sharply higher than the bimodal PTB-XL distribution centred at 74 bpm and the MIT-BIH distribution centred at 79 bpm. Table 1 confirms this with hard numbers–mean heart rate 113.5 ± 24.5 bpm in Chapman–Ningbo versus 80.4 ± 23.7 bpm in PTB-XL and 81.7 ± 18.9 bpm in MIT-BIH. The corresponding HRV indices are inversely scaled: PTB-XL has RMSSD 89.5 ± 135.3 ms and SDNN 65.1 ± 84.2 ms, while Chapman–Ningbo collapses to RMSSD 27.9 ± 96.2 ms and SDNN 22.6 ± 62.0 ms. This pattern is consistent with the higher prevalence of sinus tachycardia in the Chinese sample; because no activity or acquisition-state metadata accompany either cohort, no inference about the recording state is drawn, and the RR-derived indices are read only as 10-second surrogates whose absolute values carry the upward bias quantified in Supplementary Table S3. Figure 3D shows the lead-wise mean RMS amplitude as a heat-map for the two 12-lead cohorts; Chapman–Ningbo is uniformly 5%–15% higher in amplitude than PTB-XL across all 12 leads, with the largest absolute differences in the precordial leads V2–V4 (0.31 vs. 0.29 mV in V2). The QT duration is the only signal-level metric that is conserved across all three populations (441–443 ms), which indicates that repolarisation-timing descriptors are the most stable of the signal-level measurements considered here. This conservation should not, however, be read as the explanation for the strong LVH transfer reported below. LVH is diagnosed principally from voltage and morphology rather than from repolarisation timing, and the transferability analysis of Section 3.6 attributes its undiminished performance to a dependence on global voltage and morphology descriptors that carry small standardised mean differences across cohorts (Figure 4C); the conserved QT duration is reported here as a descriptive property of the shared signal space rather than as a mechanism of transfer.

### 3.3 Cross-population shift in the 38-D feature space

Figure 4 collapses the population-specific signal characteristics of Section 3.2 into the 38-dimensional handcrafted feature space and quantifies the resulting distributional shift. Figure 4A shows the PCA projection of the standardised pooled feature matrix; the first two components capture 25.2% and 12.6% of the variance respectively (38% cumulative). Although the three cohorts overlap substantially in the high-density centre of PC1–PC2 space, the MIT-BIH points (green) form a clearly separable elongated cluster extending towards positive PC1, echoing the acquisition gap visible in Figure 2. Figure 4B presents the non-linear UMAP projection of the same feature matrix and confirms that the cohort identity is recoverable from the handcrafted features alone: MIT-BIH occupies a distinct sub-region in the upper-right quadrant, Chapman–Ningbo concentrates in the upper-left, and PTB-XL occupies the diffuse central region. The persistence of separable cohort clusters in both linear (PCA) and non-linear (UMAP) projections is the visual counterpart of a measurable distributional gap that motivates explicit evaluation of cross-cohort transfer. Figure 4C ranks the top-15 features by the absolute standardised mean difference (SMD) between PTB-XL and Chapman–Ningbo, and the dominant axes are exactly those identified in Section 3.2: qrs_count_10s (SMD = −1.49) and hr_mean (SMD = −1.37) are markedly higher in Chapman–Ningbo, while spectral entropy (SMD =  + 1.10), pNN50 (SMD =  + 0.78), SDNN (SMD =  + 0.57) and RMSSD (SMD =  + 0.52) are markedly higher in PTB-XL. The lead-wise RMS amplitudes (lead_II, lead_aVF, lead_aVR) carry small but consistent negative SMDs (−0.34 to −0.21), mirroring the heat-map of Figure 3D. Figure 4D collapses these per-feature shifts into three pairwise MMD^2^ values computed with an unbiased estimator under an RBF kernel whose bandwidth is set per subsample by the median-distance heuristic, over 250 observations per cohort and 100 independent subsamples. The two 12-lead cohorts are close to one another (PTB-XL versus Chapman–Ningbo MMD^2^ = 0.085, 95% CI 0.074–0.099), whereas MIT-BIH lies far from both (versus PTB-XL 0.511, 95% CI 0.477–0.546; versus Chapman–Ningbo 0.500, 95% CI 0.466–0.538), and all three permutation tests reject distributional equality (*p* = 0.002). The bandwidth matters: at a fixed *γ* = 0.5 all three statistics collapse to 0.0009–0.0024, which is the resolution floor of the estimator and cannot support an ordering, so the median heuristic is used throughout and the full sweep is given in Supplementary Table S5. Figure 4E repeats the computation in the learned 64-dimensional embedding across five training seeds: the backbone reduces the gap (PTB-XL versus Chapman–Ningbo 0.060 ± 0.004; versus MIT-BIH 0.352 ± 0.014), but across those seeds a smaller embedding-space MMD is not associated with better target discrimination; the correlation is weak, of the opposite sign to the alignment hypothesis, and far from significant (Pearson r =  + 0.37, *p* = 0.54; Spearman *ρ* =  + 0.10, *p* = 0.87), as Figure 4F shows. The maximum mean discrepancy is therefore reported here as a descriptive measure of how far apart the cohorts lie, and not as the mechanism through which transfer succeeds. Table 2 summarises these findings and provides the exact mean ± SD pairs for the top-15 SMD features, allowing the magnitude of every shift to be reproduced in physical units (bpm, ms, mV).

### 3.4 Network architecture and training dynamics

**Figure 5:**
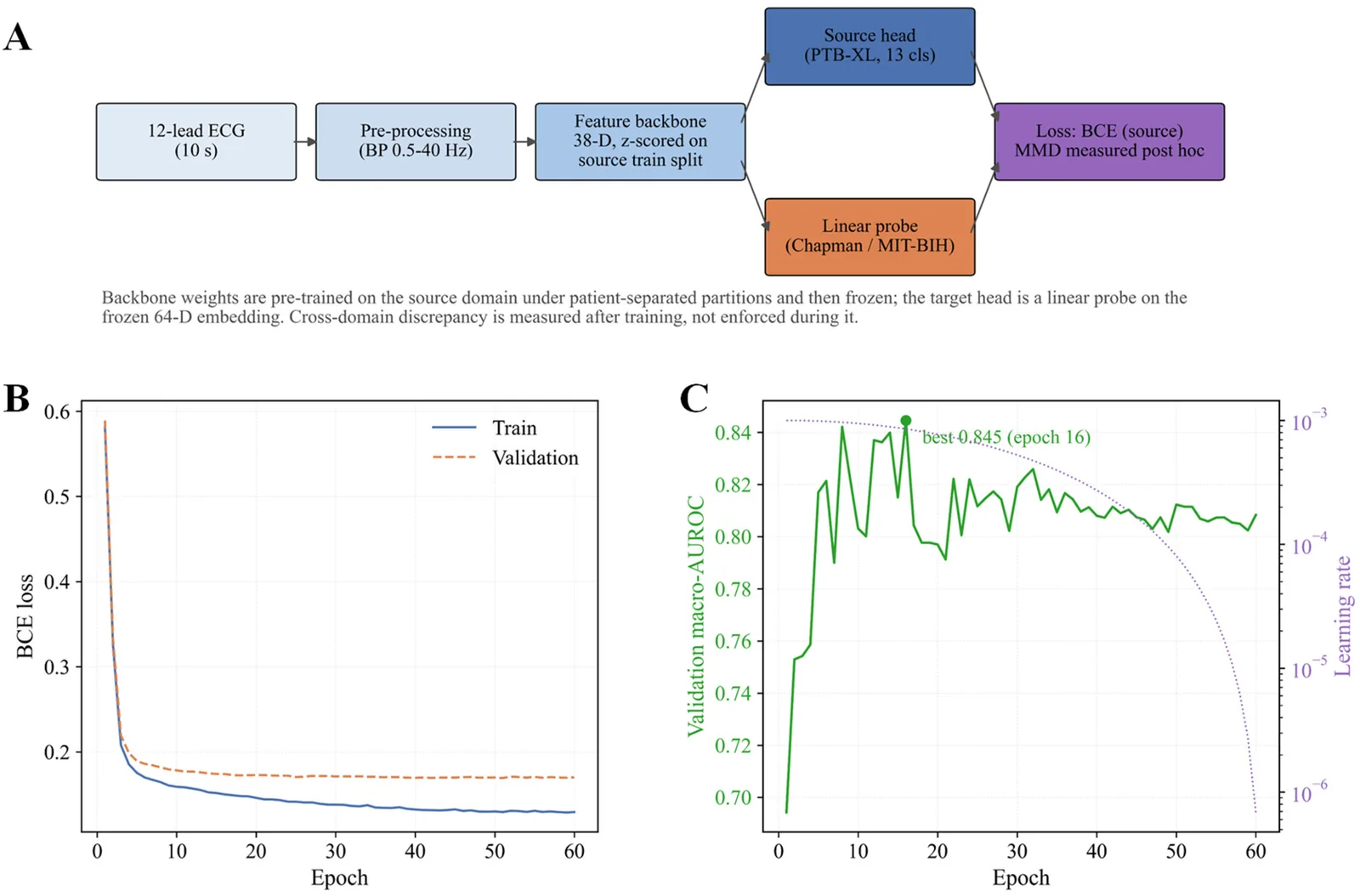
**(A)** schematic of the shared 38-D feature backbone with separate source (PTB-XL, 13 classes) and target (chapman/MIT-BIH) heads. **(B)** Per-epoch binary cross-entropy for the training and validation partitions on PTB-XL. **(C)** Validation macro-AUROC (green, left axis) and the cosine learning-rate schedule (purple, right axis); the best validation macro-AUROC of 0.845 is reached at epoch 16, but training always runs to epoch 60 and the final-epoch model is the one evaluated.

Figure 5A schematises the cross-population feature backbone described in Section 2.3. The 12-lead 10-second ECG enters the pipeline through the 0.5–40 Hz band-pass and z-score block, then through the 38-dimensional handcrafted feature backbone, which feeds two heads in parallel: a source head (PTB-XL, 13 classes) and a target head (Chapman or MIT-BIH). Training optimises the source-domain binary cross-entropy loss; the embedding-level alignment depicted in Figure 5A is reported and quantified post-hoc, not enforced as a training term–see Section 3.3 (MMD on the standardised feature space) and Section 3.9 (sensitivity sweep over an explicit MMD regulariser) for the empirical justification. Table 3 lists the architecture and training hyperparameters in full reproducible detail: the backbone contains 30,400 trainable parameters, and the complete source model contains 31,245. The model was trained for 60 epochs with AdamW (weight-decay 10^–4^, initial LR 10^–3^, cosine annealing), BCE loss only, batch size 64 and a 70/15/15 patient-grouped source split. The compactness of the model is deliberate, and the reasoning behind that choice is a design hypothesis rather than a finding of the present analysis: high-capacity CNNs operating on raw 12-lead waveforms may be over-parameterised relative to the amount of cross-population information the data actually carry, and would then be prone to over-fitting source-domain idiosyncrasies, whereas the 38-D input bottleneck explicitly factorises the cross-population shift into a small set of clinically interpretable axes (Section 3.3). No raw-waveform CNN was trained in this study, so this motivation is not tested empirically here; the comparators reported in Section 3.9 are classical models fitted on the same 38-D features, and the absence of a raw-waveform baseline is stated among the limitations in Section 4. Figure 5B shows the per-epoch BCE loss for the training and validation partitions on PTB-XL. The two curves fall together from 0.581/0.589 at epoch 0 to 0.129/0.170 at epoch 60, and the residual train–validation gap of 0.041 in binary cross-entropy is the modest degree of over-fitting that the regularisation strategy (dropout 0.2–0.3 plus weight decay) leaves in place at this model capacity. Figure 5C overlays the validation macro-AUROC (green) and the cosine learning-rate schedule (purple). Validation macro-AUROC rises steeply from 0.694 at epoch 0 to 0.817 by epoch 4 and peaks at 0.845 at epoch 16, after which it oscillates between 0.80 and 0.83 without further improvement while the cosine schedule decays the learning rate. Training runs the full 60 epochs in every configuration and no early stopping is applied, so the validation partition is used only to monitor convergence; all reported test results come from the final epoch and are therefore not selected on validation performance.

### 3.5 Source-domain class-by-class performance

**Figure 6:**
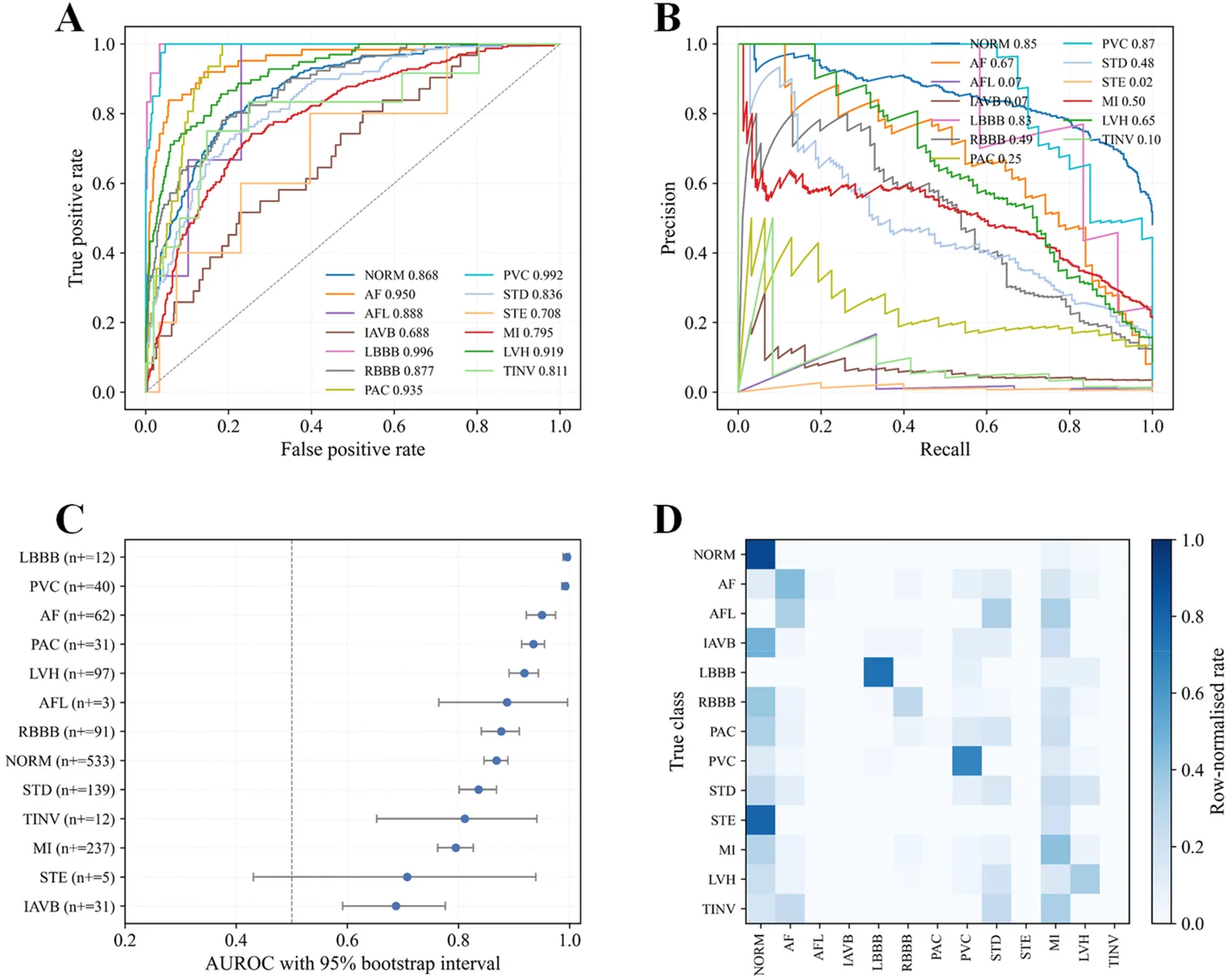
**(A)** Per-class ROC curves with AUROC. **(B)** Per-class precision-recall curves with average precision (AP). **(C)** Per-class AUROC with 95% bootstrap confidence intervals. **(D)** Row-normalised confusion matrix at the argmax prediction; entries show top-1 recall for each ground-truth class.

Figure 6 evaluates the source-trained backbone on the 1,106-record held-out test partition of PTB-XL, which shares no patient with the training or validation partitions, across all 13 unified classes. Figure 6A shows the per-class ROC curves. Five classes exceed AUROC 0.90 (LBBB 0.996 [0.988, 1.000], PVC 0.992 [0.986, 0.996], AF 0.950 [0.922, 0.975], PAC 0.935 [0.914, 0.955], LVH 0.919 [0.891, 0.944]; values in brackets are 95% non-parametric bootstrap intervals over 1,000 resamples), five lie between 0.80 and 0.90 (AFL 0.888 [0.765, 0.996], RBBB 0.877 [0.841, 0.909], NORM 0.868 [0.846, 0.889], STD 0.836 [0.801, 0.869], TINV 0.811 [0.653, 0.941]) and three fall below 0.80 [MI 0.795 [0.763, 0.827], STE 0.708 [0.431, 0.939], IAVB 0.688 [0.592, 0.776]]. The width of an interval tracks the positive count rather than the point estimate: AFL, TINV and STE have 3, 12 and 5 positive test cases respectively and their intervals span 0.23 to 0.51 AUROC, so their point estimates carry little information, whereas NORM (533 positives) and MI (237 positives) are estimated to within 0.05. Every class clears the 10-positive threshold in the training partition, so all 13 are trained and reported; the smallest are STE (13 training positives) and AFL (26). Figure 6B reports the corresponding precision-recall curves, and the contrast with the ROC ranking is informative: NORM, present in half the cohort, combines a mid-ranking AUROC with the highest average precision, while AFL combines a high AUROC with an average precision whose interval covers almost the entire unit range because there are three positives. This is why both AUROC and average precision are reported with intervals throughout. Figure 6C sorts the classes by AUROC with 95% bootstrap intervals, and Figure 6D presents the row-normalised confusion matrix at the argmax prediction, whose diagonal is the top-1 recall reported for the source domain; PVC and RBBB show a characteristic confusion with NORM because ectopic-beat segments retain a normal sinus baseline between ectopic beats. Taken together, the source-trained backbone ranks well for the high-prevalence classes and the cleanly separable rhythms, adequately for the ischaemic classes, and unreliably for the very-low-prevalence classes, and the macro-AUROC over all 13 classes is 0.866 (95% CI 0.837–0.891).

### 3.6 Cross-population transfer

**Figure 7:**
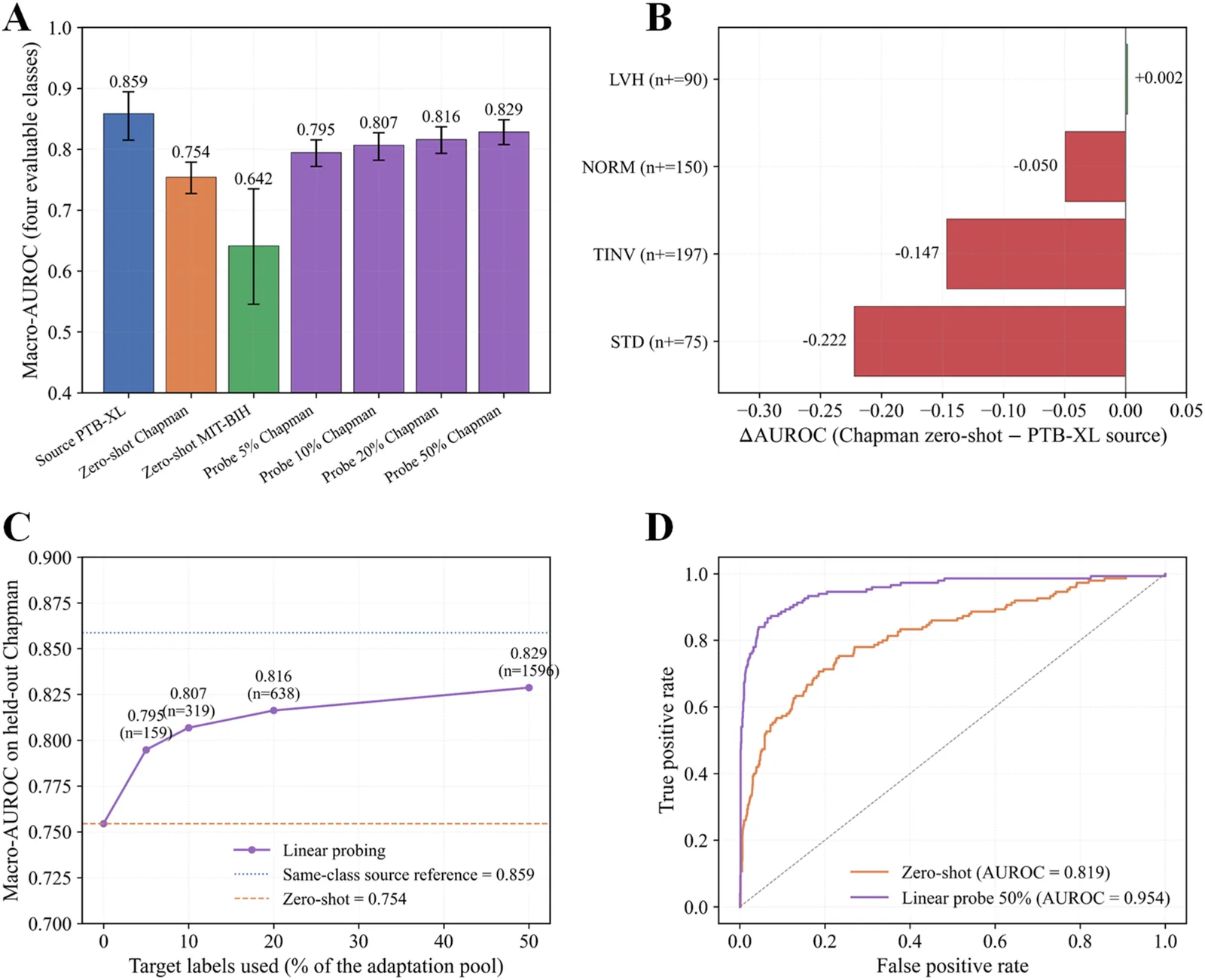
**(A)** macro-AUROC across training regimes computed on the four evaluable target classes {NORM, STD, LVH, TINV}, with 95% bootstrap intervals; cohort-specific sample sizes are annotated. **(B)** Per-class transferability gap, that is, the Chapman zero-shot AUROC net of the source AUROC, shown only for classes with at least 10 target positives. **(C)** Sample-efficiency curve for linear probing, annotated with the zero-shot value and the same-class source-domain comparator (PTB-XL macro-AUROC over the same four classes). **(D)** ROC curve for NORM on the held-out Chapman–Ningbo test set under zero-shot versus 50%-linear-probing regimes.

Figure 7 evaluates how the source-trained backbone transfers to the two target cohorts, and Figure 7A summarises the macro-AUROC of every regime side by side. A class is reported in the macro-average when the adaptation subset and the held-out test split each contain at least five positives and five negatives; this evaluability criterion governs whether a target head can be fitted and a class-level AUROC estimated at all, and it is distinct from the stricter requirement of at least 10 test positives that Section 2.6 applies before a class enters the classwise statistical-testing family. Under the prevalence-aware sampling described in Section 2.4, four of the 13 unified super-classes–NORM, STD, LVH and TINV–satisfy this condition at every label fraction including 5%, whereas sampling the adaptation subset at random satisfies it for only one class at 5%. These four classes contribute 150, 75, 90 and 197 positives respectively to the held-out Chapman test partition, so they satisfy the stricter testing threshold of Section 2.6 as well as the evaluability criterion. The remaining nine classes have at most two positive cases in the Chapman test partition and are reported in Table 6 without interpretation. The same-class comparator for every target number is therefore the source-domain macro-AUROC over exactly these four classes, 0.859 (95% CI 0.815–0.894), and not the full 13-class source figure. Zero-shot transfer to Chapman–Ningbo reaches 0.754 (95% CI 0.727–0.779), which is 88% of that comparator, indicating that the handcrafted representation generalises substantially to a different 12-lead population without any target supervision. Zero-shot transfer to MIT-BIH reaches 0.642 with a record-clustered 95% interval of 0.546–0.735, the expected consequence of the much larger acquisition gap quantified in Figure 4D. Linear probing with 5% of the Chapman adaptation pool (*n* = 159 records) already recovers most of the achievable gain (0.795, 95% CI 0.772–0.815); 10% reaches 0.807 (0.782–0.827), 20% reaches 0.816 (0.793–0.837) and 50% reaches 0.829 (0.808–0.848); the per-class values behind this curve are given in Supplementary Table S6. The increments diminish steadily–doubling the labelled data from 20 to 50% adds 0.013–and the intervals at 20% and 50% overlap, so the marginal value of further target labels is small but, unlike in the three-class analysis, adaptation does not reach the same-class source comparator of 0.859. The paired-bootstrap comparison of linear probing at 50% against zero-shot is significant after Holm correction for three of the four evaluable classes (NORM *Δ*AUROC =  + 0.135, 95% CI +0.102 to +0.171, adjusted *p* = 0.004; STD +0.093, +0.032 to +0.154, adjusted *p* = 0.012; TINV +0.065, +0.028 to +0.103, adjusted *p* = 0.004) and not significant for LVH (+0.004, −0.011 to +0.020, adjusted *p* = 0.59), the last reflecting the already-high zero-shot performance of LVH (0.920). Figure 7C plots the same macro-AUROC against the fraction of target labels used, with the zero-shot value and the four-class source comparator annotated. The convex shape of the curve indicates that the bulk of the transfer gain is achieved with very small amounts of target supervision, thereby reducing the amount of target-domain annotation required to evaluate adaptation at a new institution. Table 7 consolidates the source-domain and transfer results for the four evaluable classes together with the Holm-adjusted comparisons, and Table 6 lists the nine classes that the target cohort cannot support.

**Table 6:** The nine unified super-classes that the chapman–ningbo test partition cannot support, reported for completeness and excluded from every aggregate.

Class	*n* + source	AUROC source [95% CI]	*n* + Chapman test	Status on the target cohort
AF	62	0.950 [0.922, 0.975]	0	absent from the target cohort; not evaluable
AFL	3	0.888 [0.765, 0.996]	0	absent from the target cohort; not evaluable
IAVB	31	0.688 [0.592, 0.776]	0	absent from the target cohort; not evaluable
LBBB	12	0.996 [0.988, 1.000]	1	1 positive case; not interpretable
RBBB	91	0.877 [0.841, 0.909]	0	absent from the target cohort; not evaluable
PAC	31	0.935 [0.914, 0.955]	1	1 positive case; not interpretable
PVC	40	0.992 [0.986, 0.996]	0	absent from the target cohort; not evaluable
STE	5	0.708 [0.431, 0.939]	0	absent from the target cohort; not evaluable
MI	237	0.795 [0.763, 0.827]	2	2 positive cases; not interpretable

**Table 7:** Source-domain and cross-population transfer performance for the four evaluable target classes, with holm-adjusted paired-bootstrap comparisons of linear probing at 50% against zero-shot.

Class	*n* + source	AUROC source [95% CI]	*n* + Chapman test	Zero-shot	Linear probe 50%	*Δ*AUROC [95% CI]	p (Holm)
NORM	533	0.868 [0.846, 0.889]	150	0.819	0.954	+0.135 [+0.102, +0.171]	0.004
STD	139	0.836 [0.801, 0.869]	75	0.614	0.707	+0.093 [+0.032, +0.154]	0.012
LVH	97	0.919 [0.891, 0.944]	90	0.920	0.924	+0.004 [−0.011, +0.020]	0.593
TINV	12	0.811 [0.653, 0.941]	197	0.665	0.730	+0.065 [+0.028, +0.103]	0.004
Macro (four evaluable classes)	–	0.859 [0.815, 0.894]	–	0.754 [0.727, 0.779]	0.829 [0.808, 0.848]	–	–
Macro (All 13 Source Classes)	–	0.866 [0.837, 0.891]	–	–	–	–	–

Paired bootstrap (linear probing at 50% versus zero-shot, B = 2,000, holm-adjusted across the four evaluable classes): NORM ΔAUROC =  + 0.135 [+0.102, +0.171], *p* = 0.004; STD +0.093 [+0.032, +0.154], *p* = 0.012; TINV +0.065 [+0.028, +0.103], *p* = 0.004; LVH +0.004 [−0.011, +0.020], *p* = 0.59. Across four independent seeds with the class set held fixed: source 0.864 ± 0.005, Chapman zero-shot 0.755 ± 0.006, Chapman linear probing at 50% 0.834 ± 0.004 (mean ± SD).

The per-class transferability gaps in Figure 7B decompose the zero-shot result into ΔAUROC = AUROC(Chapman zero-shot)−AUROC(source), restricted to the four classes with sufficient positive support. LVH is the only class that transfers without loss (0.920 on Chapman versus 0.919 on the source test partition), which is consistent with its dependence on global voltage and morphology descriptors that carry small standardised mean differences in Figure 4C. NORM (0.819 versus 0.868), STD (0.614 versus 0.836) and TINV (0.665 versus 0.811) all lose discrimination, and the two largest losses are the ST-segment and T-wave classes whose absolute scale changes substantially between cohorts, so they cannot transfer without target-domain adaptation. That NORM also degrades is intuitive: a normal ECG is implicitly defined by the absence of abnormalities relative to the source population, and the heart-rate gap of Section 3.2 systematically pulls Chapman–Ningbo records out of the source-domain definition of normal sinus rhythm. The apparently high zero-shot values for LBBB, PAC and MI on Chapman–Ningbo rest on one or two positive cases each and are reported in Table 6 without interpretation; they are not evidence of positive transfer. Figure 7D plots the ROC curve for NORM on the held-out Chapman–Ningbo test set under zero-shot and 50%-linear-probing regimes; the AUROC rises from 0.819 to 0.954, the largest per-class gain attributable to adaptation observed in this study, and it motivates using adapted rather than zero-shot probabilities in the composite score of Section 3.7.

### 3.7 Composite diagnostic score

**Figure 8:**
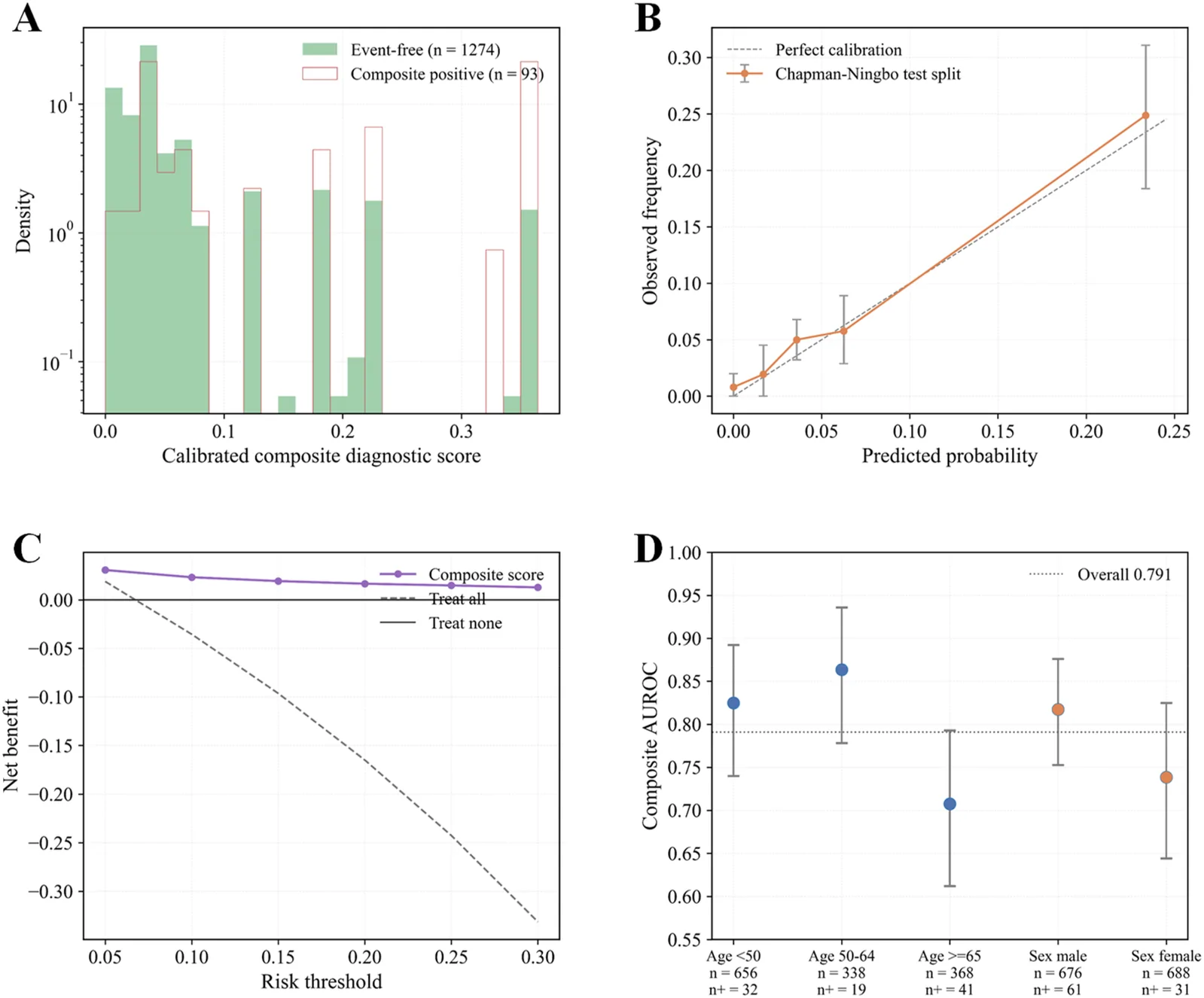
**(A)** distribution of the composite diagnostic score on the untouched chapman–ningbo test split, stratified by the composite outcome (AUROC = 0.791, 95% CI 0.743–0.836; *n* = 1,367, 93 positives of which 90 are LVH-positive). **(B)** Reliability diagram after isotonic recalibration fitted on the dedicated calibration split, with bootstrap uncertainty bands; Chapman–Ningbo Brier 0.055, expected calibration error 0.018, calibration slope 0.78. **(C)** Decision-curve analysis showing the model against treat-all and treat-none baselines across risk thresholds 0.05–0.30. **(D)** Subgroup AUROC by age stratum (< 50, 50–64, ≥ 65) and sex with 95% bootstrap intervals and per-stratum sample sizes and positive counts; the age interaction is significant (*p* = 0.012).

The adapted per-class probabilities of Section 3.6 were combined into a composite diagnostic score using a noisy-OR rule over the seven conditions {AF, AFL, MI, LBBB, RBBB, IAVB, LVH}. On the Chapman–Ningbo target only LVH has substantial positive support (90 positives; zero-shot AUROC 0.920), while LBBB and MI contribute one and two cases respectively and AF, AFL, RBBB and IAVB none at all, so 90 of the 93 composite positives on this cohort (97%) are LVH-positive and the Chapman composite is properly described as an LVH-dominated diagnostic composite rather than a broad cardiovascular-risk score. The PTB-XL source cohort has substantial support across all seven conditions (414 composite positives in the held-out test partition), so the PTB-XL composite is the demanding test of the noisy-OR formulation and attains AUROC 0.834 (95% CI 0.808–0.858); the disease-specific scores that underlie it are reported in Supplementary Table S7. Figure 8A presents the distribution of the composite score on the untouched Chapman–Ningbo test split, stratified by outcome: event-free records concentrate below 0.05 while positive records spread across the upper range, giving AUROC 0.791 (95% CI 0.743–0.836). Figure 8B presents the reliability diagram after isotonic recalibration fitted on the dedicated calibration split and applied unchanged to the test split. Calibration on genuinely untouched data is good but not perfect: Brier score 0.055, expected calibration error 0.018, calibration-in-the-large +0.009 and calibration slope 0.78, the last indicating mild over-dispersion of the predicted probabilities. Figure 8C presents the decision-curve analysis; the score delivers positive net benefit over both treat-all and treat-none across risk thresholds from 0.05 to 0.30, which indicates the range over which the score shows potential clinical utility within this retrospective cohort; because the analysis is retrospective and the Chapman composite is overwhelmingly LVH-driven, this is not evidence of demonstrated clinical benefit. Figure 8D breaks the discrimination down by demographic subgroup, and here the evidence does not support a claim of uniform performance. Discrimination ranges from 0.708 (95% CI 0.612–0.793; *n* = 368, 41 positives) in the oldest stratum to 0.863 (0.778–0.936; *n* = 338, 19 positives) in the middle stratum and 0.825 (0.740–0.892; *n* = 656, 32 positives) in the youngest, a maximum gap of 0.156 that is unlikely under exchangeability (permutation *p* = 0.037) and is accompanied by a significant score-by-age interaction (likelihood-ratio *p* = 0.012). Across sex the gap is smaller (males 0.817, 0.753–0.876, *n* = 676; females 0.738, 0.644–0.825, *n* = 688; permutation *p* = 0.116) but the calibration slopes differ materially (0.97 versus 0.44; interaction *p* = 0.015). Subgroup sample sizes, positive counts, prevalences, operating characteristics at thresholds of 0.10 and 0.20, Brier scores and calibration slopes are reported in full in Table 8, and the corresponding calibration detail in Supplementary Table S8. The composite score is therefore well calibrated overall within this target cohort but demonstrably weaker in patients aged 65 years and over, which is the stratum in which cardiovascular screening matters most.

**Table 8:** Subgroup discrimination, operating characteristics and calibration of the composite diagnostic score on the untouched chapman–ningbo test split.

Subgroup	n	n+	Prevalence	AUROC [95% CI]	Se/Sp at 0.10	Se/Sp at 0.20	Brier	Calibration slope
age <50	656	32	0.049	0.825 [0.740, 0.892]	0.50/0.92	0.38/0.96	0.040	0.83
age 50–64	338	19	0.056	0.863 [0.778, 0.936]	0.63/0.87	0.58/0.96	0.042	1.34
age >=65	368	41	0.111	0.708 [0.612, 0.793]	0.49/0.84	0.39/0.91	0.089	0.48
sex male	676	61	0.090	0.817 [0.753, 0.876]	0.61/0.86	0.49/0.94	0.067	0.97
sex female	688	31	0.045	0.738 [0.644, 0.825]	0.35/0.91	0.29/0.96	0.040	0.44

Se, sensitivity; Sp, specificity. Scores are the isotonically calibrated composite fitted on the dedicated calibration split and evaluated on the untouched test split. Pairwise contrasts, age: <50 vs. 50–64, ΔAUROC −0.039 [−0.147, +0.074]; <50 vs. >=65, ΔAUROC +0.117 [−0.008, +0.227]; 50–64 vs. >=65, ΔAUROC +0.156 [+0.035, +0.285]. Pairwise contrasts, sex: male vs. female, ΔAUROC +0.079 [−0.025, +0.192]. Likelihood-ratio interaction test on the calibration model, age *p* = 0.012, sex *p* = 0.015. Permutation test on the largest between-subgroup AUROC gap, age gap 0.156 (*p* = 0.037), sex gap 0.079 (*p* = 0.116).

### 3.8 Interpretability of the learned model

**Figure 9:**
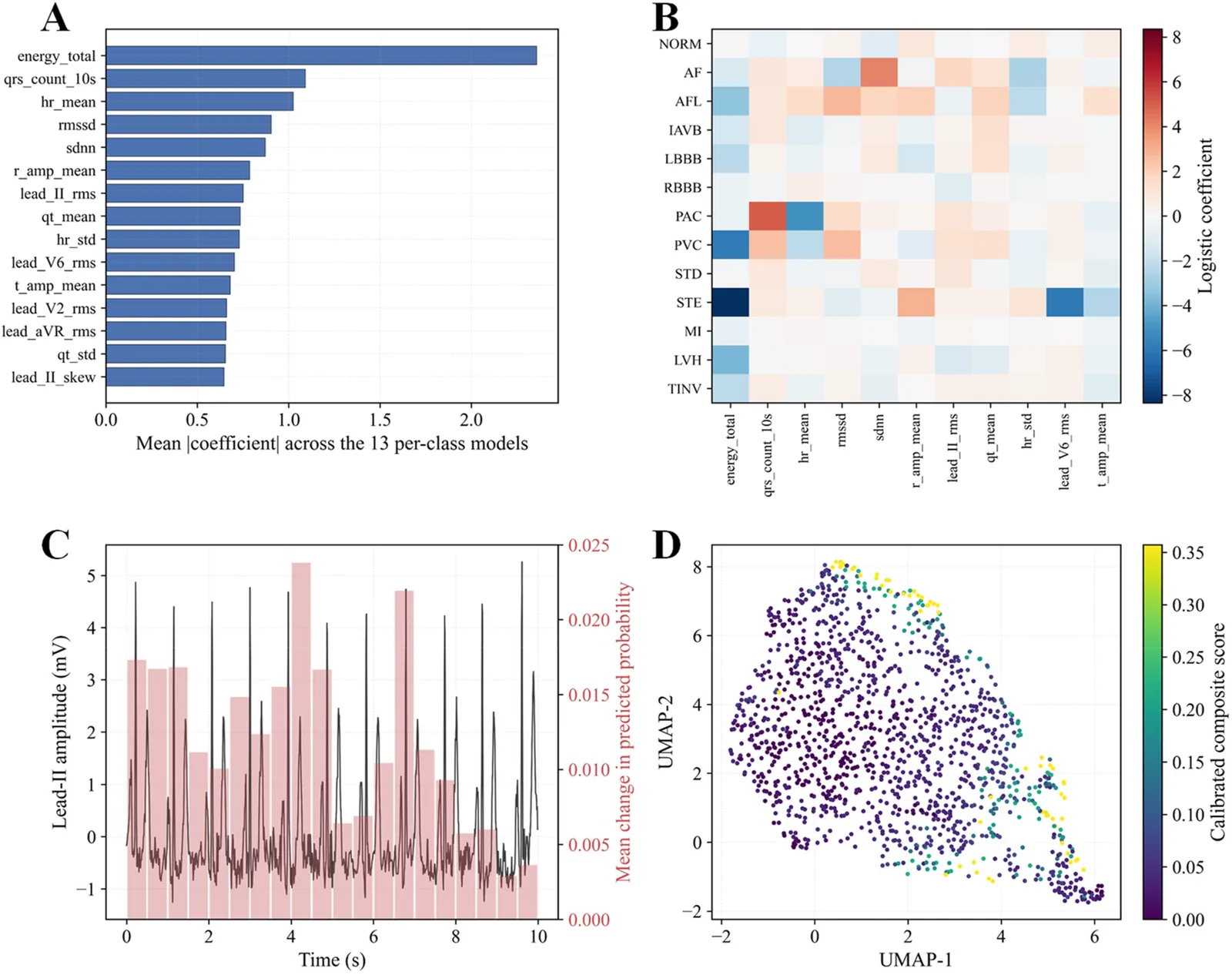
**(A)** Top-15 input features by mean |coefficient| across 13 per-class L_2_-regularised logistic regressions fitted on the standardised 38-D input features of the source training partition, with balanced class weights and at most 2,000 iterations. These are coefficients of an input-feature model and are not attributions of the neural backbone. **(B)** Class-by-feature coefficient heat-map for the top-11 features of the same input-feature model. **(C)** Window-level perturbation analysis on a representative PTB-XL record: each of 20 contiguous 0.5 s windows of the raw lead-II signal is replaced by the cohort-median level, all 38 features are recomputed on the modified signal, and the backbone is re-run; bars give the resulting mean absolute change in predicted probability across the 13 source outputs, which is not a gradient saliency map and is uncorrelated with the local amplitude of the window. **(D)** UMAP embedding of the 64-dimensional learned representation on Chapman–Ningbo, coloured by the predicted composite score.

Figure 9 examines which measurements drive the predictions, and it is important to be precise about what each panel estimates. Figure 9A and Figure 9B report the coefficients of 13 per-class L_2_-regularised logistic regressions fitted directly on the standardised 38-D input features of the source training partition; they describe the input-feature space, and they are not attributions of the neural backbone, whose internal representation is not identifiable from a downstream linear head. Read that way, the four features with the largest mean absolute coefficient are total energy (2.36), QRS density (1.09), mean heart rate (1.02) and RMSSD (0.90), which is what one would expect because these are the first-order rate and amplitude summaries that any clinically meaningful ECG classifier must encode. The per-class heat-map exposes class-specific patterns that are consistent with clinical electrocardiography: atrial fibrillation loads positively on SDNN (+4.15) and negatively on the standard deviation of heart rate (−2.65) and RMSSD (−2.44), the signature of an irregularly irregular rhythm; premature atrial contraction pairs a strong negative loading on mean heart rate (−5.08) with an equally strong positive loading on QRS density (+5.07), which is exactly how an extra ectopic complex presents in a 10-second window; and ST-elevation loads negatively on total energy (−8.35) and on the V6 root-mean-square amplitude (−5.90) while loading positively on V2 (+3.40), a precordial amplitude gradient. Figure 9C is a feature-recomputation perturbation analysis rather than a gradient saliency map: each of 20 contiguous 0.5 s windows of the raw lead-II signal is replaced by the cohort-median level, all 38 features are recomputed on the modified signal, and the backbone is re-run, so the plotted quantity is the change in predicted probability attributable to removing that window's contribution to the handcrafted features. Two things follow. The effect of removing any single window is small, between 0.003 and 0.024 in mean absolute probability change, which is the expected behaviour of a model whose inputs are aggregates over the whole segment. And the size of that effect is not explained by the local amplitude or energy of the window (correlation with the window peak amplitude r =  + 0.12; with window energy r = −0.19), so the analysis does not support a claim that the model attends preferentially to the QRS complexes; it supports the weaker and more defensible statement that no single half-second of the recording dominates the prediction. Figure 9D embeds the 64-dimensional learned representation of the Chapman–Ningbo records in a 2-D UMAP space coloured by predicted composite score, and shows a smooth gradient from the low-score to the high-score region, indicating that the score varies continuously over the embedding rather than as a noisy patchwork.

### 3.9 Robustness, baselines and sensitivity analyses

We complement the headline transfer numbers of Section 3.6 with three robustness analyses addressing single-seed reporting, the absence of strong baselines and the *post-hoc* treatment of cross-domain alignment. First, because re-running a pipeline under seeds that perturb the partition and the initialisation at the same time confounds two different quantities, we separate them. Holding the patient-grouped partition fixed and varying only the initialisation across four seeds gives a source macro-AUROC of 0.864 ± 0.005, a Chapman zero-shot four-class macro-AUROC of 0.755 ± 0.006 and a linear-probing 50% value of 0.834 ± 0.004. Holding the initialisation fixed and drawing ten repeated patient-grouped partitions gives 0.844 ± 0.018, 0.774 ± 0.023 and 0.838 ± 0.017 respectively. Sampling variability is therefore three to four times larger than optimisation variability on every quantity, and the confidence with which any single split can be reported is governed by the former; Table 9 and Supplementary Table S9 report both. Enforcing patient-level grouping changes the source macro-AUROC from 0.857 to 0.866 and the Chapman zero-shot value from 0.761 to 0.754, both well inside the sampling standard deviation, so the grouping is methodologically necessary but not outcome-determining.

**Table 9:** Separation of optimisation variability from sampling variability under patient-grouped partitioning.

Analysis	What is held fixed	Source macro (13 classes)	Chapman zero-shot (4 classes)	Chapman 50% adaptation (4 classes)
Primary analysis	patient-grouped partition, seed 42	0.866	0.754	0.829
Optimisation variability (SD)	partition fixed; 4 initialisation seeds	± 0.005	± 0.006	± 0.004
Sampling variability (SD)	initialisation fixed; 10 repeated patient-grouped partitions	± 0.018	± 0.023	± 0.017
Record-level source split	seed 42, patients allowed to span partitions	0.857	0.761	0.832

Second, we benchmarked the backbone against two classical models trained on exactly the same 38-D features, splits and standardisation, with no neural representation: a per-class L_2_-regularised logistic regression and a Random Forest (200 trees, maximum depth 10). On the source PTB-XL test partition the backbone leads across the full 13-class label space (0.866 versus 0.826 for logistic regression and 0.816 for the Random Forest), and on Chapman zero-shot transfer it leads over the four evaluable classes by 0.016 to 0.029 macro-AUROC (0.754 versus 0.738 and 0.725; Table 10). The advantage is present in both regimes and is largest where it matters clinically, in cross-population transfer, which is consistent with the non-linear projection extracting a representation less sensitive to the population-specific shifts of Section 3.3 than the input features themselves. We note that this is an observation about relative performance under a fixed feature set and not a demonstration that the backbone has learned an aligned representation, a claim the MMD analysis of Section 3.3 does not support.

**Table 10:** Classical baselines fitted on the same 38-D features, splits and standardisation, with no neural representation.

Model	Source macro (13 classes)	Chapman zero-shot (4 classes)	Chapman linear probe 50% (4 classes)
Logistic regression (C = 0.5)	0.826	0.738	–
Random Forest (200 trees, depth 10)	0.816	0.725	–
Proposed backbone (this work)	0.866	0.754	0.829

Third, we investigated whether explicitly enforcing cross-domain alignment during training would improve transfer. We added an MMD regulariser *λ* ∈ {0, 0.01, 0.1, 1.0} between the source-batch and Chapman-mini-batch 64-D embeddings during pre-training, with all other settings held constant. Across the four values the source macro-AUROC ranged from 0.854 to 0.866, the Chapman zero-shot four-class macro-AUROC from 0.742 to 0.754, and the linear-probing 50% value from 0.818 to 0.838 (Table 11). The spread at *λ* ≤ 0.1 lies within twice the multi-seed standard deviation, so those configurations are statistically indistinguishable from the baseline, while the strongest setting *λ* = 1.0 is worse on every metric. This is a deliberate negative result: for this feature pipeline at this scale, an explicit alignment term adds nothing that the shared scaler, the batch-normalisation layers and the harmonised preprocessing do not already provide. We therefore report the simpler BCE-only training as the main pipeline and present the *λ* sweep as its empirical justification.

**Table 11:** Sensitivity of training to an explicit MMD-alignment regulariser λ.

λ	Source macro (13 classes)	Chapman zero-shot (4 classes)	Chapman linear probe 50% (4 classes)
0.00	0.866	0.754	0.829
0.01	0.864	0.754	0.838
0.10	0.864	0.753	0.836
1.00	0.854	0.742	0.818

Finally, because freezing the backbone is a modelling choice rather than a necessity, we compared the three adaptation regimes defined in Section 2.4 on the same splits (Table 12). Unfreezing the backbone helps only when target labels are plentiful. At 50% of the target labels, full fine-tuning reaches a four-class macro-AUROC of 0.850 against 0.828 for partial fine-tuning and 0.829 for linear probing, a gain of +0.021 over the primary regime; at 5% the ordering reverses and linear probing leads (0.795 against 0.778 for full and 0.750 for partial fine-tuning), which is the behaviour predicted for feature distortion under distribution shift when the adaptation set is small (40). Calibration favours linear probing at every budget: the expected calibration error at 50% labels is 0.017 for linear probing against 0.030 for full and 0.036 for partial fine-tuning, and at 5% labels the gap widens to 0.035 against 0.132 and 0.134. Linear probing is therefore retained as the primary regime because it is the best-calibrated option at every label budget, the only one that is competitive in the small-label regime this study targets, and the cheapest, updating 260 parameters rather than 31,245; full fine-tuning is preferable when discrimination alone is the objective and at least half the target cohort can be labelled. This comparison also fixes the terminology: the primary protocol of this study is linear probing, and the term fine-tuning is reserved for the two regimes that actually update backbone weights.

**Table 12:** Adaptation regimes compared on identical splits: discrimination and expected calibration error over the four evaluable classes.

Target labels	Records	Linear probing (primary) AUROC/ECE	Partial fine-tuning AUROC/ECE	Full fine-tuning AUROC/ECE
5%	159	0.795/0.035	0.750/0.134	0.778/0.132
10%	319	0.807/0.025	0.768/0.120	0.803/0.114
20%	638	0.816/0.019	0.789/0.083	0.825/0.077
50%	1,596	0.829/0.017	0.828/0.036	0.850/0.030
Updated parameters	–	260	9,229	31,245

AUROC is the macro-average over {NORM, STD, LVH, TINV}; ECE is the mean expected calibration error over the same four classes. Linear probing updates the per-class heads only; partial fine-tuning additionally unfreezes the final backbone block; full fine-tuning updates every parameter.

## 4 Discussion

This study characterises the cross-population structure of the electrocardiographic feature space, the model that operates on it, and the composite diagnostic score that emerges from it. Four findings deserve particular emphasis. First, the population-specific shift in the 38-D handcrafted feature space is dominated by two axes–heart rate and QRS density–with absolute standardised mean differences of 1.37 and 1.49 respectively, while amplitude-related and morphological features carry an order of magnitude smaller shifts. This dichotomy has direct methodological implications: a network trained end-to-end on raw waveforms is exposed to every source of variation simultaneously and may expend disproportionate capacity on the rate axis, whereas a handcrafted-feature backbone separates the rate axis from the morpholog*y* axis by construction. The compactness of our backbone (30,400 trainable parameters, three orders of magnitude smaller than canonical ECG CNNs) is therefore a substantive design choice and not merely a computational convenience, although the present data cannot separate its contribution from that of the feature pipeline it sits on.

Second, adaptation is strikingly label-efficient but does not close the gap to the same-class source comparator. Linear probing with 5% of the target adaptation pool (159 records) reaches a four-class macro-AUROC of 0.795% and 50% reaches 0.829, against a source-domain four-class reference of 0.859, so roughly four-fifths of the achievable gain is obtained from the first 159 labelled recordings while the remainder is not recovered by more labels of the same kind. This is a regime that a target institution can realistically supply. The paired-bootstrap comparison shows that the gain over zero-shot survives Holm correction for NORM, STD and TINV but not for LVH, which already transfers with no measurable loss. The overall picture is that adaptation is essential for the ST-T-driven classes and unnecessary for the voltage-driven one, and that prevalence-aware sampling of the adaptation subset is what makes the smaller label budgets usable at all.

Third, the proposed backbone outperforms two classical baselines fitted on the same features, splits and standardisation, both on the source domain (0.866 versus 0.826 and 0.816) and on cross-population zero-shot transfer (0.754 versus 0.738 and 0.725), while an explicit MMD alignment term adds nothing over BCE-only training. It is tempting to read this as implicit alignment, and the earlier version of this analysis did so, but the evidence does not support that reading. The backbone does reduce the measured discrepancy between cohorts (input-space MMD^2^ 0.085 falling to 0.060 in the learned embedding for PTB-XL versus Chapman–Ningbo), yet across five training seeds a smaller embedding-space discrepancy is not associated with better target discrimination (r =  + 0.37, *p* = 0.54). We therefore report the performance advantage as an empirical observation and the discrepancy measurements as a description of how far apart the cohorts lie, without asserting a causal link between them.

Fourth, the recalibrated composite diagnostic score retains useful discrimination (AUROC 0.791 on the untouched Chapman test split, 0.834 on the seven-condition PTB-XL composite) and good calibration (Brier 0.055, expected calibration error 0.018, calibration slope 0.78), but its performance is not uniform across patients. Discrimination in the oldest stratum is 0.708 against 0.863 in the middle stratum, a gap of 0.156 supported by both a permutation test (*p* = 0.037) and a score-by-age interaction test (*p* = 0.012), and the calibration slope varies from 0.44 in women to 1.34 in the middle age stratum. An earlier reading of similar point estimates as evidence of demographic robustness was not supportable: similar AUROCs across strata are not equivalence, and once subgroup sizes, positive counts, intervals and calibration are reported together the heterogeneity becomes visible, which is exactly the failure mode that audits of clinical machine-learning models are designed to surface (41). We also emphasise that on Chapman–Ningbo the composite is dominated by LVH detection (97% of positives), so its value there is best read as a calibrated, transferable LVH detector, while the PTB-XL composite–built on a substantially more diverse high-risk class mix–is the more demanding test of the noisy-OR formulation as a multi-condition integrator.

Compared with prior published cross-population ECG work, our results are competitive and, we would argue, more completely reported. Table 13 sets the present study beside recent multi-dataset transfer, domain-generalisation, self-supervised representation and external-validation studies along the dimensions that determine whether a cross-population claim can be checked: the datasets and target populations used, how the label spaces were harmonised, the adaptation strategy and model size, the fraction of target labels consumed, and whether calibration, subgroup performance and external validation were reported at all. Recent reports on PTB-XL alone have placed source-domain macro-AUROC for the ten super-classes between 0.85 and 0.92 with end-to-end CNN architectures at 10^6^–10^7^ trainable parameters (21); our 0.866 (95% CI 0.837–0.891) across the larger 13-class label space is within the same range despite using a 30-thousand-parameter backbone on 38 handcrafted features, consistent with the wider observation that in-domain accuracy and transferability are only loosely coupled (42); contrastive pre-training on cardiac signals reports linear-evaluation gains of a comparable magnitude on dataset-native label sets (43). The PhysioNet/Computing in Cardiology Challenge 2020/2021 quantified the cross-database AUROC drop on heterogeneous 12-lead collections and reported zero-shot losses in the 0.05–0.15 range across model families (44), a range consistent with our 0.105 drop (0.859 to 0.754) on the four evaluable classes. What Table 13 shows is not that the present framework outperforms these studies, but that the combination of calibration on an independent split, subgroup analysis with interval estimates and formal interaction tests, and an explicit statement of how many target labels were consumed, is rarely reported together; that combination, rather than any single performance number, is the contribution we claim. The companion PTB-XL + data release extends the source benchmark with additional features and labels and suggests that the same evaluation protocol could be applied to a richer label space without altering the present methodology (45).

**Table 13:** The present study set beside recent cross-population and transfer-learning electrocardiographic work along the dimensions that determine whether a cross-population claim can be checked.

Study	Datasets/target populations	Label harmonisation	Adaptation strategy	Model size	Target labels used	Calibration reported	Subgroup analysis	External validation	Reported performance
Strodthoff et al. 2021 (21)	PTB-XL (Germany); internal folds only	PTB-XL SCP super-classes (5–71 labels)	supervised training, no cross-population adaptation	10^6^–10^7^ parameters	not applicable	no	no	no (internal folds)	macro-AUROC 0.85–0.93 in-domain
Reyna et al. 2021 (37)	12 databases across 4 continents	SNOMED-CT challenge scoring vocabulary	per-team; mostly joint multi-source training	varies, mostly 10^6^–10⁸	full labels of all training sources	no	no	yes (hidden test sets)	cross-database AUROC drop 0.05–0.15
Mehari and Strodthoff 2022 (12)	PTB-XL, Chapman, Ribeiro (self-supervised)	dataset-native label sets, not unified	self-supervised pre-training then supervised fine-tuning	10^6^–10^7^ parameters	full target label set	no	no	partial (cross-dataset fine-tuning)	macro-AUROC 0.87–0.94 after fine-tuning
Kiyasseh et al. 2021 (39)	four public ECG datasets	dataset-native label sets	contrastive pre-training, linear evaluation	10^6^ parameters	1%–100% in linear evaluation	no	no	no	AUROC gains of 0.02–0.10 over supervised
Kumar et al. 2022 (43)	vision and language benchmarks (methodological)	not applicable	linear probing versus full fine-tuning under distribution shift	varies	varies	no	no	yes (OOD test sets)	linear probing can beat fine-tuning out-of-distribution
This work	PTB-XL (Germany) → Chapman–Ningbo (China), MIT-BIH (USA)	13 unified SNOMED-CT super-classes, full mapping in Supplementary Table S2	linear probing (primary); partial and full fine-tuning compared	31,245 source-model parameters (30,400 backbone)	5%, 10%, 20%, 50% (159–1,596 records)	yes: independent calibration split, slope, ECE, Brier, all with intervals	yes: age and sex, with intervals, operating points and interaction tests	yes: two held-out target cohorts, zero-shot and adapted	source 0.866; target zero-shot 0.754; linear probe 50% 0.829 (4 classes)

Several limitations should be acknowledged. First, the target evaluation rests on four of the 13 unified classes; prevalence-aware sampling widened the evaluable set from three classes to four, but nine classes still have at most two positive cases in the Chapman test partition and are reported without interpretation. Extending the statistical basis requires a target cohort with adequate positive support across atrial fibrillation, myocardial infarction and conduction block–a large hospital-scale resource such as MIMIC-IV-ECG (46) would be the natural next target–and not merely a different split of the present one. Second, the composite endpoint is diagnostic rather than prospective: it is defined at the moment of acquisition, no follow-up data exist for these cohorts, and on Chapman–Ningbo it is dominated by LVH; it should not be read as a cardiovascular-event risk model. Third, discrimination and calibration differ across age strata and calibration differs across sex, so the score is not yet suitable for unmodified deployment in older patients, and subgroup-specific recalibration is a necessary next step. Fourth, the study uses cohorts from public databases collected at single institutions in each country and is therefore not representative of national populations; Chapman–Ningbo's high prevalence of T-wave inversion and elevated heart-rate distribution in particular may reflect an institution-specific clinic mix. Fifth, the MIT-BIH cohort has a fundamentally different acquisition protocol and its 576 segments derive from only 48 recordings; it is evaluated zero-shot only, all its intervals are record-clustered, and it serves as a stress test rather than a primary target. Sixth, the 10-second RR-derived features are segment-level surrogates with a quantified upward bias against reference annotations and are not standard short-term heart-rate-variability measurements. Seventh, the noisy-OR composite assumes class-conditional independence, and a multi-class calibration such as Dirichlet recalibration might extract additional discrimination. Finally, no raw-waveform deep-CNN baseline was run, and all validation is retrospective and internal to the target cohort; prospective evaluation in a real screening setting has not been performed. Future work should extend the framework to additional cohorts, incorporate longitudinal follow-up to validate the score against hard outcomes, and investigate whether foundation-model embeddings of raw waveforms can replace the handcrafted features.

## 5 Conclusions

A compact 38-dimensional, clinically interpretable feature backbone with 30,400 parameters, pre-trained with binary cross-entropy on the German PTB-XL cohort under patient-separated partitions, transfers to a Chinese target cohort with a four-class macro-AUROC of 0.754 (95% CI 0.727–0.779) without any target supervision, and reaches 0.795 (0.772–0.815) with 5% and 0.829 (0.808–0.848) with 50% of target labels by linear probing on the frozen embedding, against a same-class source-domain reference of 0.859; unfreezing the backbone adds 0.021 at the largest label budget but costs calibration and loses its advantage when few target labels are available. The composite diagnostic score derived from the adapted model, recalibrated on a dedicated calibration split and evaluated on untouched test data, attains AUROC 0.791 with a Brier score of 0.055 and an expected calibration error of 0.018, and shows positive net benefit across decision thresholds from 0.05 to 0.30 in retrospective decision-curve analysis, which indicates potential rather than demonstrated clinical utility. Its discrimination is, however, materially lower in patients aged 65 years and over (0.708 versus 0.863 in the middle stratum, interaction *p* = 0.012), and on the Chinese cohort the endpoint is dominated by left-ventricular hypertrophy. Interpretable and label-efficient cross-population ECG models are therefore achievable without large end-to-end networks, and this study provides a reproducible template for building and–equally important–for auditing them; extending evaluable-class coverage, correcting the age-related performance gap, and prospective validation in real screening settings remain necessary before clinical use.

## Data Availability

Publicly available datasets were analyzed in this study. This data can be found here: the data and code that support the findings of this study are openly available on Zenodo at https://doi.org/10.5281/zenodo.21193806.
