# Supplementary material for: Label-efficient cross-population transfer learning for electrocardiographic risk stratification: evaluation across German, Chinese and US cohorts
Source: Front Cardiovasc Med. 2026 Sep 10;13:1928354. doi: 10.3389/fcvm.2026.1928354 (PMC13601223; doi:10.3389/fcvm.2026.1928354)
Supplement: Supplementary file 1 [file Table1.docx]

**Supplementary Material**

Supplementary Table S1. Cohort construction, grouping unit, and patient and record counts.

| **Cohort** | **Records / segments** | **Distinct patients or recordings** | **Grouping unit enforced in all partitions** |
| --- | --- | --- | --- |
| PTB-XL (Germany) | 7371 | 6643 patients (544 with more than one record, covering 1272 records) | patient_id |
| Chapman–Ningbo (China) | 4574 | 4574 records, one per subject | record |
| MIT-BIH (USA) | 576 | 48 source recordings (12 segments each) | source recording |
| Inclusion path | a record is included when (i) its WFDB header and signal files are present, (ii) the 38-D feature vector is finite in every component, and (iii) its native diagnostic codes map into the unified vocabulary; records whose codes map to no super-class are retained as all-negative |  |  |

Supplementary Table S2. Complete native-to-super-class label mapping and the ambiguity rules applied.

| **Unified super-class** | **PTB-XL SCP codes** | **Chapman SNOMED-CT codes** | **MIT-BIH beat symbols** |
| --- | --- | --- | --- |
| NORM | NORM | — | — |
| AF | AFIB | — | — |
| AFL | AFLT | — | — |
| IAVB | 1AVB | — | — |
| LBBB | CLBBB, ILBBB | — | — |
| RBBB | CRBBB, IRBBB | — | — |
| PAC | PAC | — | — |
| PVC | PVC | — | — |
| STD | STD_, DIG, ISC_, ISCAN, ISCAL, ISCAS, ISCIN, ISCIL, ISCLA | — | — |
| STE | STE_ | — | — |
| MI | IMI, AMI, LMI, PMI, ASMI, ILMI, ALMI, INJAS, INJAL, INJIN, INJIL, INJLA | — | — |
| LVH | LVH | — | — |
| TINV | INVT | — | — |
| Ambiguity rule 1 | labels are retained in multi-label form; a record may map to several super-classes and is never collapsed to a single class |  |  |
| Ambiguity rule 2 | isolated rate-only findings (sinus tachycardia 427084000, sinus bradycardia 426177001) are deliberately NOT mapped to NORM, because the rate is already carried by the hr_mean feature |  |  |
| Ambiguity rule 3 | a record whose native codes match no entry of the translation table is kept in the cohort with an all-zero label vector |  |  |
| Ambiguity rule 4 | the source head is trained on every super-class with at least 10 positives in PTB-XL, which is all 13 (smallest: STE = 13, AFL = 26) |  |  |

Supplementary Table S3. Validation of the QRS detector and of the 10-second RR-derived indices against the MIT-BIH reference beat annotations.

| **Quantity** | **Value** |
| --- | --- |
| QRS detection sensitivity (150 ms tolerance) | 0.997 |
| QRS detection positive predictive value | 0.965 |
| True positives / false positives / false negatives | 7,260 / 265 / 20 |
| Mean heart rate (bpm): reference vs detector | r = 0.716; bias +3.6; 95% limits of agreement -23.0 to +30.2 |
| RMSSD (ms): reference vs detector | r = 0.871; bias +23.2; 95% limits of agreement -148.7 to +195.1 |
| SDNN (ms): reference vs detector | r = 0.860; bias +17.3; 95% limits of agreement -92.7 to +127.3 |

Supplementary Table S4. MIT-BIH zero-shot performance with record-clustered intervals and the record-level repeat of the analysis (576 segments from 48 recordings).

| **Class** | **Segment-level AUROC** | **Naive 95% CI** | **Record-clustered 95% CI** | **Record-level AUROC [95% CI]** |
| --- | --- | --- | --- | --- |
| NORM | 0.476 | [0.426, 0.526] | [0.339, 0.614] | 0.531 [0.360, 0.704] |
| LBBB | 0.607 | [0.545, 0.668] | [0.433, 0.786] | 0.705 [0.497, 0.887] |
| RBBB | 0.552 | [0.440, 0.663] | [0.148, 0.942] | 0.633 [0.288, 0.913] |
| PAC | 0.744 | [0.653, 0.825] | [0.501, 0.890] | 0.558 [0.337, 0.776] |
| PVC | 0.829 | [0.788, 0.865] | [0.741, 0.906] | 0.731 [0.572, 0.877] |
| Macro | 0.642 | — | [0.546, 0.735] | 0.631 |

Supplementary Table S5. Maximum mean discrepancy under different kernel bandwidths, in the input and the learned embedding space, with the test of association between embedding-space alignment and target discrimination.

| **Configuration** | **PTB-XL vs Chapman** | **PTB-XL vs MIT-BIH** | **Chapman vs MIT-BIH** |
| --- | --- | --- | --- |
| Input space, fixed γ = 0.05 | 0.0871 | 0.1706 | 0.2073 |
| Input space, fixed γ = 0.1 | 0.0448 | 0.0540 | 0.0746 |
| Input space, fixed γ = 0.5 | 0.0009 | 0.0020 | 0.0024 |
| Input space, fixed γ = 1.0 | 0.0001 | 0.0008 | 0.0008 |
| Input space, median-distance heuristic | 0.0848 | 0.5112 | 0.5000 |
| Learned embedding, median heuristic (mean ± SD over 5 seeds) | 0.0600 ± 0.0044 | 0.3519 ± 0.0136 | 0.3642 ± 0.0186 |
| Permutation test against the cohort-label-shuffled null (median heuristic, 500 permutations) | p = 0.002 | p = 0.002 | p = 0.002 |
| Association with target discrimination across 5 training seeds | Pearson r = +0.370 (p = 0.540); Spearman ρ = +0.100 (p = 0.873) |  |  |

Supplementary Table S6. Per-class label-efficiency curve for linear probing under prevalence-aware sampling of the adaptation subset.

| **Target labels** | **Records** | **NORM** | **STD** | **LVH** | **TINV** | **Macro (4 classes) [95% CI]** |
| --- | --- | --- | --- | --- | --- | --- |
| 5% | 159 | 0.921 | 0.641 | 0.924 | 0.693 | 0.795 [0.772, 0.815] |
| 10% | 319 | 0.943 | 0.677 | 0.901 | 0.706 | 0.807 [0.782, 0.827] |
| 20% | 638 | 0.945 | 0.688 | 0.910 | 0.721 | 0.816 [0.793, 0.837] |
| 50% | 1596 | 0.954 | 0.707 | 0.924 | 0.730 | 0.829 [0.808, 0.848] |
| Zero-shot | 0 | 0.819 | 0.614 | 0.920 | 0.665 | 0.754 [0.727, 0.779] |

Supplementary Table S7. Disease-specific scores underlying the seven-condition PTB-XL composite.

| **Condition** | **n+ (PTB-XL test)** | **AUROC [95% CI]** | **Brier** | **Calibration slope** |
| --- | --- | --- | --- | --- |
| AF | 62 | 0.950 [0.922, 0.975] | 0.029 | 0.86 |
| AFL | 3 | too few positives to evaluate | — | — |
| MI | 237 | 0.795 [0.763, 0.827] | 0.135 | 0.93 |
| LBBB | 12 | 0.996 [0.988, 1.000] | 0.004 | 1.27 |
| RBBB | 91 | 0.877 [0.841, 0.909] | 0.056 | 1.05 |
| IAVB | 31 | 0.688 [0.592, 0.776] | 0.027 | 0.71 |
| LVH | 97 | 0.919 [0.891, 0.944] | 0.049 | 0.94 |
| Composite (noisy-OR over all seven) | 414 | 0.834 [0.808, 0.858] | 0.157 | 0.99 |

Supplementary Table S8. Calibration of the composite diagnostic score on the untouched test split, overall and within every demographic subgroup.

| **Subgroup** | **n** | **n+** | **Brier** | **ECE** | **Calibration-in-the-large** | **Calibration slope** | **Calibration intercept** |
| --- | --- | --- | --- | --- | --- | --- | --- |
| age <50 | 656 | 32 | 0.040 | 0.006 | -0.000 | 0.83 | -0.42 |
| age 50-64 | 338 | 19 | 0.042 | 0.027 | -0.005 | 1.34 | +0.56 |
| age >=65 | 368 | 41 | 0.089 | 0.041 | +0.037 | 0.48 | -0.67 |
| sex male | 676 | 61 | 0.067 | 0.023 | +0.020 | 0.97 | +0.24 |
| sex female | 688 | 31 | 0.040 | 0.012 | -0.003 | 0.44 | -1.50 |
| Overall (untouched test split) | 1367 | 93 | 0.055 | 0.018 | +0.009 | 0.78 | -0.32 |

Supplementary Table S9. Effect of patient-level grouping and the separation of optimisation from sampling variability.

| **Analysis** | **Source macro (13 classes)** | **Chapman zero-shot (4 classes)** | **Chapman 50% adaptation (4 classes)** |
| --- | --- | --- | --- |
| Record-level source split (as originally reported) | 0.857 | 0.761 | 0.832 |
| Patient-grouped source split (revised primary) | 0.866 | 0.754 | 0.829 |
| Optimisation variability (SD over 4 initialisation seeds) | ± 0.005 | ± 0.006 | ± 0.004 |
| Sampling variability (SD over 10 grouped partitions) | ± 0.018 | ± 0.023 | ± 0.017 |
